# Insights into the formation and diversification of a novel chiropteran wing membrane from embryonic development

**DOI:** 10.1186/s12915-023-01598-y

**Published:** 2023-05-04

**Authors:** Neal Anthwal, Daniel J. Urban, Alexa Sadier, Risa Takenaka, Simon Spiro, Nancy Simmons, Richard R. Behringer, Chris J. Cretekos, John J. Rasweiler, Karen E. Sears

**Affiliations:** 1 Department of Ecology and Evolutionary Biology, University of California, Los Angeles, USA; 2 Centre for Craniofacial and Regenerative Biology, King’s College London, London, UK; 3 Carl R. Woese Institute for Genomic Biology, University of Illinois at Urbana-Champaign, Champaign, USA; 4 Department of Mammalogy, Division of Vertebrate Biology, American Museum of Natural History, New York, USA; 5 Department of Molecular, Cell, and Developmental Biology, UCLA, Los Angeles, USA; 6 Zoological Society of London, London, UK; 7 Department of Genetics, University of Texas MD Anderson Cancer Center, Houston, USA; 8 Department of Biology, Idaho State University, Pocatello, USA; 9 Department of Obstetrics and Gynecology, State University of New York Downstate Medical Center, New York, USA

**Keywords:** Bats, Evolutionary developmental biology, Plagiopatagium, Wing membranes, Periderm

## Abstract

**Background:**

Through the evolution of novel wing structures, bats (Order Chiroptera) became the only mammalian group to achieve powered flight. This achievement preceded the massive adaptive radiation of bats into diverse ecological niches. We investigate some of the developmental processes that underlie the origin and subsequent diversification of one of the novel membranes of the bat wing: the plagiopatagium, which connects the fore- and hind limb in all bat species.

**Results:**

Our results suggest that the plagiopatagium initially arises through novel outgrowths from the body flank that subsequently merge with the limbs to generate the wing airfoil. Our findings further suggest that this merging process, which is highly conserved across bats, occurs through modulation of the programs controlling the development of the periderm of the epidermal epithelium. Finally, our results suggest that the shape of the plagiopatagium begins to diversify in bats only after this merging has occurred.

**Conclusions:**

This study demonstrates how focusing on the evolution of cellular processes can inform an understanding of the developmental factors shaping the evolution of novel, highly adaptive structures.

**Supplementary Information:**

The online version contains supplementary material available at 10.1186/s12915-023-01598-y.

## Background

Understanding how complex, novel traits emerge and diversify is an important goal of the field of evolutionary developmental biology (evo-devo) [1]. Over the last 30 years, the field has correlated genetic variation with phenotypic evolution [2–7]. Based on these efforts, researchers have proposed that novel traits mainly arise through the duplication, modification, or co-option of pre-existing patterns and processes at all biological levels [8]. For example, the novel eye spot on the butterfly wing is thought to have evolved through the co-option of gene regulatory network modules originally involved in processes such as wing patterning, healing, and appendage development [8–12] Other traits, including the horns of beetles and maxillipeds of crustaceans, are text-book examples of novelties that data suggest evolved via the divergence of serial homologs [13–15]. However, because many studies of novel traits have been performed either on an individual species and/or lack an ecological context (but see [13, 16]), we are far from having a broad, general understanding of the processes that govern origination and diversification of novel structures within and among clades. Additional studies in large, diverse taxonomic groups are needed to decipher these developmental and evolutionary mechanisms.

**Fig. 1:**
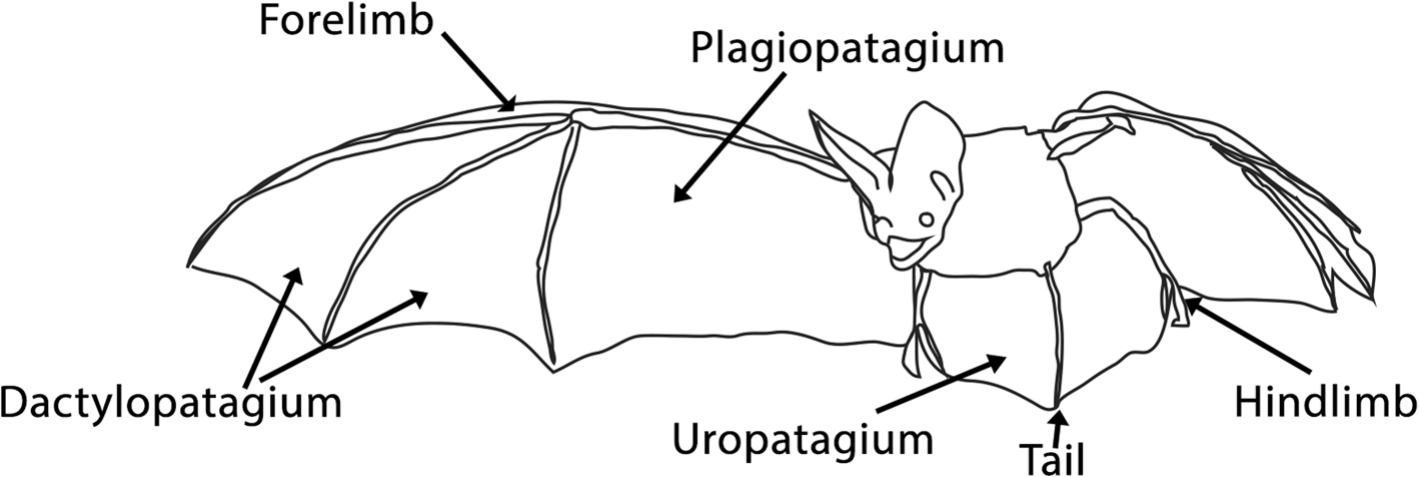
Schematic representation of the major parts of the bat wing

The wing membranes of bats—a diverse clade of over 1400 living species—provide a good model system for undertaking such studies. Bat wing membranes, or patagia, include the dactylopatagium running between the forelimb digits, the propatagium between the neck and first digit, the plagiopatagium between the caudal margin of the fore- and hind limbs, and the uropatagium between the hind limbs and tail (Fig. 1). Of these, the pro-, plagio-, and uropatagia do not have apparent homologs in other animals. While the winglets of gliding mammals and wings of birds display subsets of these membranes, these were independently acquired and are, therefore, homoplastic rather than homologous to the pro-, plagio-, and uropatagia of bats [17]. The paleontological origins of novel bat wing membranes also remain mysterious; wing membranes were clearly fully formed in the oldest fossilized bat skeletons from the early Eocene, ~ 52.5 million years ago [18–20], and no transitional fossil forms between bats and their non-volant ancestors have been uncovered.

Since their origins > 50 million years ago, the novel membranes that comprise bat wings have diversified in shape and function in tandem with the radiation of bats into a broad range of ecological niches, foraging regimes, roosting behaviors, reproductive modes, etc. [21, 22]. For example, today’s extant bat species have evolved a great diversity of diets encompassing the diets seen across Mammalia (e.g., arthropods, fishes, small vertebrates, fruits, nectar, pollen, leaves, and blood). This dietary diversity is associated with a broad range of foraging strategies and flight styles, which are, in turn, associated with significant variation in wing membrane shape. For example, the plagiopatagium, whose shape affects flight maneuverability [22, 23], tends to be broader in species that forage in dense clutter (e.g., most frugivores) and narrower in taxa that forage in open spaces (e.g., many insectivores that hunt flying insects). The same logic applies to species that tend to roost in cluttered or more open spaces [24, 25]. Roosting behaviors can exert selective pressures on bat wing morphology; case in point, several unrelated bat groups (e.g., *Myzopoda aurita*, *Eudiscopus denticulus*, *Thyroptera *sp.,* Tylonycteris pachypus*) have independently evolved suction pads on their wrists and ankles (e.g., [26–28]). For some bats, reproductive mode also impacts flight mechanics and, presumably, wing shape. For example, mothers of several bat groups carry their babies, which are frequently born at a third of the mother’s mass, while flying and feeding [25, 29]. Predator avoidance also has the potential to exert selective pressures on bat wings and biomechanics [30]. Finally, the uropatagia also displays functionally significant phenotypic diversity; many highly acrobatic insectivorous bats have large uropatagia that extend beyond the tail [22, 31, 32], while uropatagia are greatly reduced or absent in many plant-feeding species and all vampire bats.

Study of patagial development in bats is facilitated by the emergence of bats as a non-traditional mammalian model system for the study of the evolution of developmental processes. Studies of bat development have revealed that the bat wing skeleton is patterned through modulation of the limb developmental program (e.g., [20, 33–49]). Studies of the development of the dactylopatagium (the membrane between bat fingers) suggest that it is formed, at least partially, through loss of the interdigital apoptosis that characterizes hand development in most tetrapods [36, 50–52]. However, development of the novel bat wing membranes, including that of the plagiopatagium, has received relatively little attention. Anatomical descriptions of bat prenatal development, which often incorporate wing patagia, have been published as part of staging guides or broader studies for less than 1% of the 1400 + living bat species ( [53]): *Hipposideros arminger* [54], *H. pratti* [54], *Rhinolophus ferrumequinum* [55], *Rousettus aegyptiacus *[56],* R. amplexicaudatus* [57], *Syconycteris australis* [58] from the Yinpterochiroptera, and *Artibeus obscurus* [59], *A. fimbriatus* [59], *A. lituratus* [59], *Carollia perspicillata* [60], *Miniopterus natalensis* [61], *M. schreibersii* [54], *Mops condylurus* [62], *Myotis albescens* [63], *M. myotis* [64], *Molossus rufus* [65], *Pipistrellus abramus* [66], and *Vespertilio sinensis* [67] from the Yangochiroptera. To our knowledge, only one study to date has focused on the molecular or cellular development of the wing patagia, a study of patagial-associated muscle development in *Miniopterus fuliginosus* [68].

We here begin to explore two topics that are foundational to our understanding of the initiation and diversification of the bat plagiopatagium: first, when and how is the plagiopatagium initially formed during bat embryonic development (e.g., initiation), and second, when and how the plagiopatagium diversifies during development in diverse bat species (e.g., diversification). While we acknowledge that these are massive and distinct topics whose complete understanding will require additional future studies of developmental processes, gene networks, selective pressures, and adaptive variation to state but a few integral areas, this study nevertheless provides important insights and lays a foundation for years of additional research.

To begin to address plagiopatagial initiation and diversification, we combined histological assays, geometric morphometrics, scanning electron microscopy, and studies of cellular processes to characterize the developing plagiopatagium. For our morphometric studies, we used a range of bat species with distinct dietary and foraging preferences and wing shapes. While we acknowledge that multiple factors presumably influence the evolution of bat wing shape (e.g., roosting, foraging, feeding, and reproductive behavior), in this study, we solely focus on the relationships between dietary preferences, foraging activities, and the development of wing shape. Other factors, and the interplay between them, should be explored in future studies. Our findings suggest that plagiopatagium initiation is similar across bat species and that plagiopatagium shape subsequently diversifies during later growth. To gain further insights into the initiation of the patagia in bats, we used RNA-Seq to characterize the gene expression profile of two bat species with diet/foraging-related differences in plagiopatagial shape: the insectivorous *Pteronotus quadridens* and the omnivorous *Erophylla sezekorni*. Through this, we identified a subset of differentially expressed genes during plagiopatagial development in both species, including several genes with known roles in limb and epithelial development. These differentially expressed genes include *Ripk4*, a gene whose disruption has been implicated in human popliteal pterygium syndromes [69–73]. Finally, we used immunofluorescent techniques to characterize the processes by which the expanding plagiopatagium merges with the forelimb. These results suggest that the processes driving this merger include critical changes in epidermal epithelium development. These changes are like those observed during ectopic patagial formation in some other mammals [72–74].

## Results

### Initial formation of the plagiopatagium in bats

**Fig. 2:**
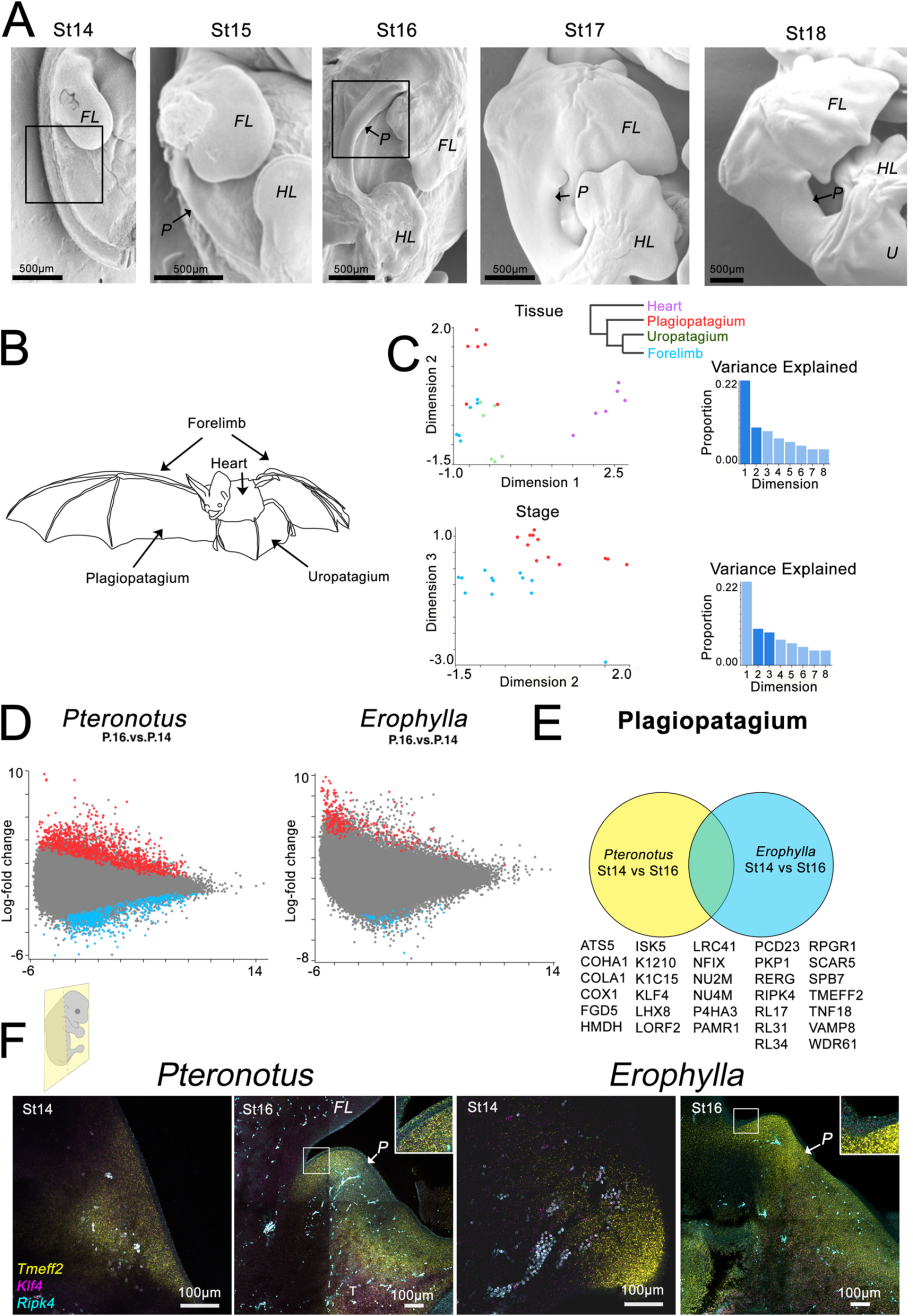
Bat patagia development is under the control of genes known to regulate epidermal epithelium. **A** Scanning electron microscopy right lateral images of the trunk region in *Carollia*, showing the development of the bat wing from St14. **B**, **C** Multidimensional scaling (MDS) analysis of gene expression showing clustering of each tissue (**B**) and developmental stage (**C**). **D** Differential expression between St14 and St16 of plagiopatagia in each species sampled. **E** Common differentially expressed genes in both *Pteronotus* and *Erophylla*. **F** HCR In situ hybridization of Tmef2, Ripk4, and Kfl4 in *Pteronotus* and *Erophylla* at St14 and St16. Boxes in **A** indicates region shown in **F**. FL—forelimb; HL—hindlimb; P—plagiopatagium; U—uropatagium

We first characterized the timing of the morphological initiation of plagiopatagium development in sixteen bat species. Sampled species include those that feed heavily (but by no means exclusively [75]) on insects, nectar, and/or fruit (Table 1 and Additional File 1: Fig. S1) and which have different wing proportions. Cross-species and cross-stage comparisons suggest that the timing of the initial outgrowth of the plagiopatagium is similar across all species. We did not detect an incipient plagiopatagium in any species at Stage (St) 14. However, by St 15, we observed an outgrowth of the future plagiopatagium along the lateral aspect of the trunk, caudal to the forelimb bud (arrow in Additional File 1: Fig. S1), in all species. We further observed that the plagiopatagium has merged to the fore- and hind limbs in all species by St 16 (Fig. 2A).

**Table 1:** Development of plagiopatagium in 16 species of bats

Family	Species	Diet	Foraging habitat	Plagiopatagium outgrowth observed	Plagiopatagium fusion
Phyllostomidae	*Artibeus jamaicensis*	Fruit, insects, pollen, leaves	Clutter	St15	St16
Phyllostomidae	*Carollia perspicillata*	Fruit, nectar, pollen, insects, leaves	Clutter	St15	St16
Phyllostomidae	*Erophylla sezekorni*	Fruit, nectar, pollen, insects	Clutter	St15	St16
Phyllostomidae	*Glossophaga soricina*	Nectar, pollen, fruit, floral parts, insects	Clutter	St15	St16
Hipposideridae	*Hipposideros larvatus*	Insects	Edge	St15	St16
Phyllostomidae	*Macrotus waterhousii*	Insects	Clutter	St15	St16
Miniopteridae	*Miniopterus australis*	Insects	Edge	St15	St16
Molossidae	*Molossus molossus*	Insects	Open space	St15	St16
Noctilionidae	*Noctilio albiventris*	Insects and rarely small fish	Over water	St15	St16
Vespertilionidae	*Pipistrellus papuanus*	Insects	Edge	St15	St16
Phyllostomidae	*Platyrrhinus helleri*	Fruit, insects	Clutter	St15	St16
Mormoopidae	*Pteronotus quadridens*	Insects	Clutter	St15	St16
Rhinolophidae	*Rhinolophus lepidus*	Insects	Open space	St15	St16
Vespertilionidae	*Rhogeessa minutilla*	Insects	Open space	St15	St16
Phyllostomidae	*Sturnira erythromos*	Fruit	Clutter	St15	St16
Pteropodidae	*Synconycteris australis*	Nectar, pollen, fruit	Clutter	St15	St16

Diet is listed with primary content of diet first. References for diets and foraging habitat in Additional File 2: Table S1

We next used RNA-Seq as a tool to identify genes expressed during plagiopatagial development. As we observed the initial outgrowth of the plagiopatagium by St 15, we inferred that developmental programs with roles in the initiation of plagiopatagial outgrowth should be activated by around St 14 and those for fusion by St 16. We, therefore, focused our RNA-Seq inquiries on St 14 and St 16. To identify genes enriched in the process of initial plagiopatagium development across bats with differing adult ecologies and plagiopatagial phenotypes, we focused on two species: *Pteronotus quadridens*, an insectivore with a relatively narrow plagiopatagium and* Erophylla sezekorni*, an omnivore with a relatively broad plagiopatagium. For each of these species, we extracted and sequenced mRNA from plagiopatagial, uropatagial, forelimb, and heart tissues at St 14 and St 16. Glimma multidimensional scaling (MDS) analysis shows that the gene expression profiles of each tissue tend to form separate clusters (Fig. 2B), as do the tissues taken from each developmental stage (Fig. 2C). However, one caveat to these assays is that the plagiopatagial tissues targeted from St 14 are best described as incipient plagiopatagial tissues rather than fully formed plagiopatagial tissues, whose possibly non-homologous tissue types might introduce some error to St 14 and St 16 comparisons.

We then compared gene expression in St 14 and St 16 in *Pteronotus quadridens* and in *Erophylla sezekorni* (Fig. 2D, E). While in this initial study, we narrowed our studies to these two taxa because of their availability and different foraging paradigms; future research would benefit from reproduction of these assays in additional, morphologically diverse bats. We identified 1037 differentially expressed genes (both up- and downregulated) in *Pteronotus quadridens* and 242 *in Erophylla sezekorni* (Fig. 2D). We then compared these two sets of differentially expressed genes and identified 32 differentially expressed genes during plagiopatagium development in both *Pteronotus quadridens* and *Erophylla sezekorni* (Fig. 2E). Genes that are differentially expressed in both species similarly are more likely to have roles in this shared process. The 32 identified genes include *Ripk4*, a regulator of periderm development in the epidermal epithelium that has been implicated in human popliteal pterygium syndromes [69, 70], *Klf4*, a classical stem cell factor that also regulates epidermal periderm development [76–79], *Tmeff2*, which was previously identified as having a role in bat limb elongation [80], and *Adamts5*, a metalloprotease that has been implicated in interdigitation [81].

Expression of *Tmeff2*, *Ripk4*, and *Klf4* was confirmed by HCR in situ hybridization in *Pteronotus* and *Erophylla* sections (Fig. 2F and Additional File 1: Fig. S2). At St 14, *Tmeff2* expression is restricted to the mesenchyme of the trunk, while at St 16, it is also expressed in the epithelium of the plagiopatagium (Fig. 2F and Additional File 1: Fig. S2). *Ripk4* is not detected a St 14, while at St 16 is expressed in both the epithelium and mesenchyme (Fig. 2F and Additional File 1: Fig. S2). *Klf4* is weakly expressed in the trunk mesenchyme at St 14 and with increased intensity at St 16 (Fig. 2F and Additional File 1: Fig. S2).

**Fig. 3:**
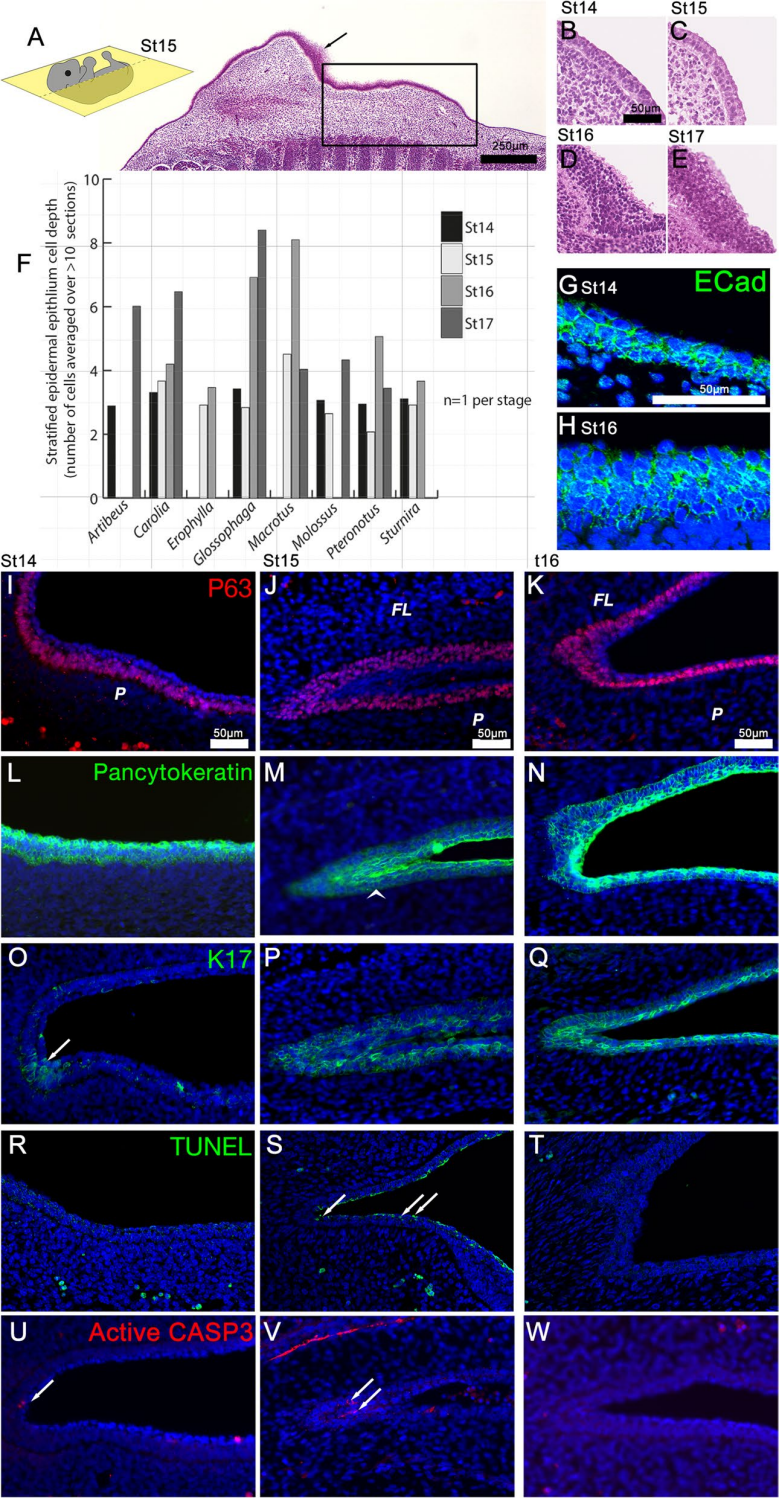
Fusion of plagiopatagium and limb epithelium. **A–E** Hematoxylin and eosin staining of *Carollia* sections showing plagiopatagium epithelium. Arrow in **A** indicates stratified epithelia in limb, at St15. Box in **A** highlights plagiopatagium. **F** Stratification of plagiopatagium across species. The mean number of stratified cells was counted for each image from a minimum of 10 basal–apical cell stacks. The mean was then calculated across three to five sections. **G, H** E-cadherin expression in *Carollia* plagiopatagium at St14(G) and St16(H). **I–T** Protein localization in *Carollia* in plagiopatagium epithelium between St14 and St16 using antibodies against P63 (**I–K**), pan cytokeratin (**L–N**), and Keratin 17 (**O–Q**). Arrowhead in **M** indicates epithelial seam formed by merging of plagiopatagium and forelimb, arrow in **O** indicates initial K17 expression at the region joining the limb to the plagiopatagium. **R–T** TUNEL apoptosis assay in *Carollia* plagiopatagium between St14 and St16. Arrow indicate positive TUNEL staining. **U–W** Active Caspase 3 (CASP3) immunofluorescence apoptosis assay in *Carollia* plagiopatagium between St14 and St16. Arrows indicate positive staining. All sections are in the coronal plane. FL—forelimb; P—plagiopatagium

Given our identification of epithelial development genes including *Ripk4* and *Klf4* in our RNA-Seq screen, we next investigated development of the epidermal epithelium. We first used hematoxylin and eosin (H&E) staining to visualize the formation of the epidermal epithelium and quantify its stratification during plagiopatagium development in eight diverse bat species (Fig. 3 and Additional File 1: Fig. S3). At St 14 and St 15, the developing epidermis of the plagiopatagium presents as a two to three cell-layer epithelium in all species (Fig. 3A–F). Basal layers are cuboidal or columnar in morphology, while the apical layer is flattened and more squamosal. At St 15, the epithelium of the plagiopatagium is becoming more stratified in the region where the plagiopatagium and the forelimb will merge. The epithelium near the fusion site is also highly stratified (Fig. 3A arrow). At St 16, the epithelium of the plagiopatagium is highly stratified, strikingly so in *Macrotus*, *Glossophaga*, and *Pteronotus* (Fig. 3D, F, Additional File 1: Fig. S3). The level of epidermal stratification remains high in the St 17 plagiopatagium (Fig. 3F).

We next performed a series of immunofluorescence assays (IF) to characterize the developing epithelium’s structure further. IF was carried out in *Carollia perspicillata* samples from the Zoological Society of London due to the limited availability of tissues from wild-caught bats. These samples were collected, fixed, and stored in optimal laboratory conditions, and so were the best available for IF. As a species, *Carollia perspicillata* has the added advantage of being arguably the most well-described model for bat development [34, 39, 51, 60, 82].

To confirm the epithelial identity of the stratified epidermis in the developing plagiopatagium, we performed IF for E-Cadherin, a cell–cell adhesion molecule and epithelial hallmark. At St 14 and St 16, we observed positive Ecad localization in all epithelial cell layers (Fig. 3G, H). We next used IF to examine the localization of P63 in *Carollia*. P63 marks the basal cells of stratified epidermal epithelia, which give rise to the apically stratified layers during development (Fig. 3I–K). Of note, we found that P63-positive cells are either stratified or pseudostratified at St 14 and St 15 in *Carollia*, both in the developing plagiopatagium and the inferior aspect of the forelimb (Fig. 3I, J). In contrast, P63 positive basal cells are not stratified or pseudostratified at St 16 except for those cells located in the region in which the plagiopatagium and limb are merging (Fig. 3K). We also used pan cytokeratin (cytokeratin 1–20) IF to visualize the level of epithelial keratinization (Fig. 3L–N). We found that keratins are present in all layers at all examined stages of plagiopatagium development.

Mice and humans with mutations in *Ripk4* display disruptions in periderm formation that result in epithelial adhesion and the generation of patagium-like structures [69–73]. Because of this, we next used IF to examine the localization of the periderm cytokeratin K17 in the developing epidermal epithelium of *Carollia* (Fig. 3O–Q). At St 14, K17-positive cells are largely absent from the epithelium of the region of the trunk that will give rise to the plagiopatagium (Fig. 3O). However, we did observe some K17-positive cells in the region where the forelimb meets the trunk (arrow Fig. 3O). At St15, when the epithelia of the forelimb and plagiopatagium are beginning to merge, K17-positive cells are present in both tissues in the region where the merging is occurring (Fig. 3P). However, K17 localization is restricted to the basal and suprabasal layers of the periderm; K17-positive cells are not present in the outermost layers of the peridermal epithelia of the forelimb or plagiopatagium. At St 16, K17 localization has expanded to include the outermost layer of peridermal epithelial cells (Fig. 3Q).

Finally, we observed a cellular remnant of the forelimb-plagiopatagium merge (i.e., an epithelial seam) at St 15 that disappeared by St 16 (arrowhead in Fig. 3M compared to Fig. 3N). To determine if the merged epidermal epithelia are being cleared by programmed cell death, i.e., apoptosis, we performed both IF for activated (cleaved) Caspase 3 and TUNEL assays on sectioned tissues from *Carollia*. We did not observe any TUNEL staining in St 14 or St 16 epithelia (Fig. 3R, T). However, TUNEL-positive cells are present in the apical-most layer of the epidermis at St 15 (Fig. 3S). Scant active caspase 3 signal was detected in the epithelia of S14 and St16 samples, while at St 15, perinuclear staining is observed at the epithelium where the trunk and forelimb merge (Fig. 3U–W).

### Diversification of plagiopatagium morphology among bats

**Fig. 4:**
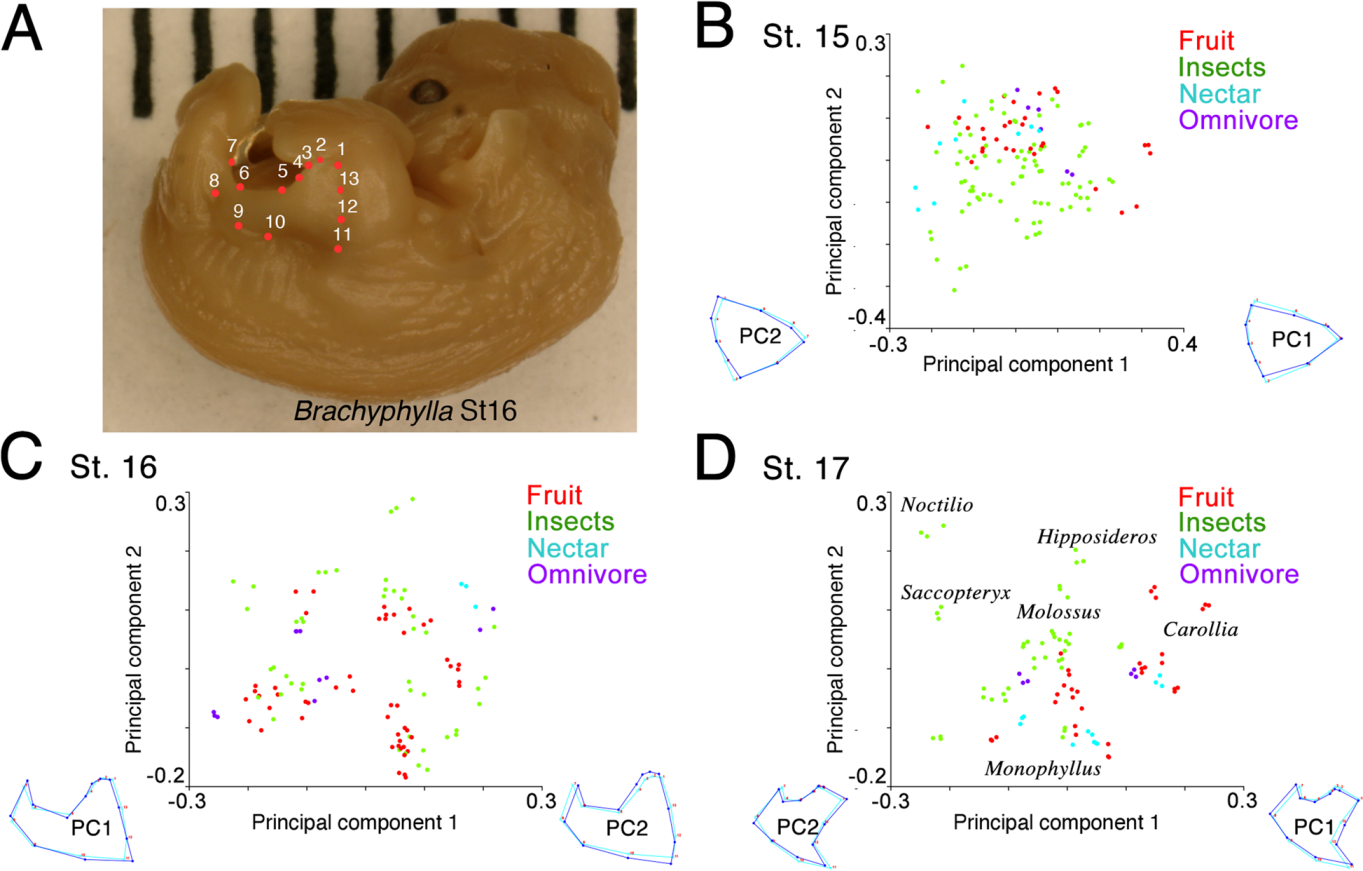
Species-specific changes in plagiopatagial growth. **A** Landmark locations demonstrated on a St16 *Brachyphylla*. **B**–**D** PCA plots of plagiopatagium morphospace for St15-17, plots contain a range of samples from 15 to 20 genera per stage, with a minimum of 3 replicates of each. Datapoints color-coded by presumed primary dietary component

From the narrower plagiopatagia of open space-foraging, insect-feeding bats to the broader ones of clutter-foraging, fruit-feeding species, plagiopatagial shape and dietary niche vary in tandem across bats in a reflection of foraging style [22, 31, 32]. To explore the developmental origins of this shape variation, we used a geometric morphometric approach to compare plagiopatagial shape across 37 diverse bat species at St 15, St 16, and St 17 (Fig. 4). We were able to examine more species for these assays than for histology because our geometric morphometric approach is non-destructive (i.e., embryos remain intact after analysis). The sampled bats include species that feed on a wide range of diets and in open, edge, or narrow/clutter environments (see Additional File 2: Table S1 for details of diet and foraging habitat). At St 15 and St 16, the stages immediately following initial plagiopatagial outgrowth and fusion, morphospace results suggest that the shape of the developing plagiopatagium is roughly similar across all sampled bats (Fig. 4B,C, Additional File 1: Fig. S4). In line with this, Kruskal–Wallis tests contrasting foraging groups did not significantly distinguish principal component 1 (PC1) medians at St 15 (chi-square = 3.48, *p* = 0.18) or St 16 (chi-square = 2.66, *p* = 0.27). However, by St 17 the plagiopatagia of bats with differing dietary preferences and foraging behaviors have begun to separate in morphospace (Fig. 1D, Additional File 1: Fig. S4). At this stage, plagiopatagial shape overlaps in clutter-foraging, plant-feeding specialists (e.g., fruit- and nectar-feeders such as *Carollia* and *Monophyllus*) but is more distinct in open- and edge-foraging, insect-feeding bats (such as *Molossus* and *Hipposideros*). Broadly, insect-feeding bats that forage in open and edge habitats occupied the more negative PC1 and positive PC2 morphospace, while clutter-foraging, plant-feeders and omnivores occupy the more positive PC1 and negative PC2 morphospace (Fig. 1D, Additional File 1: Fig. S4). The most negative PC1 values are observed in *Noctilio* and *Saccopteryx* and the most positive in *Carollia* (Fig. 4D). Consistent with this, a Kruskal–Wallis test contrasting foraging groups was able to significantly distinguish PC1 medians at St 17 (chi-square = 34.94, *p* < 0.0001).

## Discussion

This study attempts to unite molecular, cellular, and morphological to take foundational steps toward an improved understanding of the developmental basis of two critical events in bat evolution, the initial formation and morphological diversification of a novel structure, the plagiopatagium wing membrane.

Our examinations of the morphology of the developing wings of sixteen bat species indicate that the plagiopatagium consistently begins its outgrowth by St 15 and merges with the fore- and hind limbs by St 16. These sixteen species span nine families and the Yangochiroptera and Yinpterochiroptera, the two major clades of living bats, and more than double the number of species and families for which data were previously available on this subject (Table 1). Previously available data, which are consistent with the results of this study, include isolated morphological descriptions of development in six bat species from four families (*Carollia perspicillata*,* Miniopterus schreibersii*,* Hipposideros armiger*,* H. pratti*,* Molossus rufus*, and *Pipistrellus abramus* [54, 60, 65, 66]). Taken together, these results suggest that the timing of the initial physical outgrowth of the plagiopatagium and its subsequent merging to the limbs displays little to no variation (i.e., heterochrony) across bat species despite differences in adult wing shape, and thus appears highly conserved across Chiroptera.

Although there are undoubtedly many developmental processes that contribute to the initial outgrowth of the bat plagiopatagium and its subsequent merging to the limbs, results of our RNA-Seq assays provide clues into some of the processes that likely contribute to the latter of these important events. We did not find any candidates for the outgrowth of the patagium. This is likely a result of our experimental design; more targeted research will be needed to explore these events further. Importantly, our RNA-Seq dataset should be compared with data derived from comparable tissue in non-bat species, such as mouse, to identify any patagium specific programs. This is an acknowledged limitation of this current study.

The above limitations notwithstanding, our data set provides some candidates for patagium formation of relevance to the merging of the plagiopatagium and limbs; our RNA-Seq assays identified multiple genes as being differentially expressed in the developing plagiopatagia of both *Pteronotus quadridens* and *Erophylla sezekorni*. Of the genes identified in this study, only one, *Tmeff2*, was previously associated with bat limb development [80]. The remaining genes include several with crucial roles in epithelial development, including *Ripk4*. *Ripk4* is a downstream target of P63, a master regulator of ectodermal stratification [76, 83]. Disruption of *Ripk4* in humans can result in popliteal pterygium syndrome [69, 70], a condition in which abnormal skin fusions can occur under the arms and between the legs. Mouse knockout studies indicate that *Ripk4* is required for proper epidermal epithelium formation; its loss generates both a thickened epidermal epithelium and a lack of periderm. As a result, limb and flank epithelial tissues meet without the protective periderm—the “non-stick” layer of the epidermal epithelium [77]—and merge.

Our histological and IF results in the bat *Carollia perspicillata* suggest that the processes regulating the merging of the plagiopatagium and limb in bats might be analogous to those regulating the merging of the flank and limb in *Ripk4*-deficient humans and mice. Like the phenotype in *Ripk4*-disrupted mice, the epithelium of the *Carollia* plagiopatagium, including the P63-positive basal cells, becomes increasingly stratified during development. In addition, in *Carollia*, as in *Ripk4*-disrupted mice, the plagiopatagium/flank and limbs meet and merge without a definitive layer of K17-expressing periderm. In contrast, the epithelium of wild-type mice at similar stages appears simple, with an apical-most cell layer marked by K17 expression [69]. These findings suggest that changes in bat plagiopatagium formation might involve the P63-dependent development of the epithelium. In addition, we also observed apoptotic cell death in the outermost layer of the developing plagiopatagium and limb in St 15 *Carollia* during the merging of these tissues. This finding suggests a possible role of programmed cell death in helping prevent the formation of a mature apical periderm in pre-fusion plagiopatagia and limbs (see [77, 84, 85] for similar processes in other organs). Given the morphological similarity of early plagiopatagium development across bat species, it is possible or even probable that our results in *Carollia* will translate across bats. However, future research in more bat species is needed to test this possibility.

In addition to flank-limb fusions, *Ripk4*-deficient mice commonly display orofacial clefting [77]. Intriguingly, bats also show a high propensity for orofacial clefting, which has been proposed to be linked to their use of echolocation. Non-pathological palate clefting normally occurs in about half of all living bat species. The anterior skull’s cleft structure is a normal part of craniodental morphology in these taxa [86–89]. The prevalence of non-pathological palate clefting in bats raises the possibility that a common regulatory mechanism underpins the development of the wing membranes and cleft palates of bats. Whether bats evolved flight or echolocation first is still debated, although a “flight-first” hypothesis is likely [19]. Therefore, it may be that the regulatory changes that drove the evolution of novel wing membranes may have also played a permissive role in the evolution of non-pathological palate clefting in bats. Developmental exploration of palatal clefting across bats is needed to test this hypothesis.

Following the plagiopatagium-limb merger, the resulting epithelium seam between the limb and plagiopatagium is likely removed through cellular processes such as migration, type-transition, and/or programmed death. Migration-led remodeling has been linked to the removal of epithelial seams during mouse palate development [77] and digit separation [84], although both these processes also rely on coordinated programmed cell death. In *Carollia*, we only observed a small number of cleaved Caspase 3-positive cells after the merging of the plagiopatagium and limb in bats, suggesting that apoptosis may not be the only process by which the epithelial seam between these tissues is cleared.

Our histological and IF results suggest that the earliest plagiopatagium outgrowth stages up to and including its merging to the limb are highly conserved among bats with diverse foraging behaviors and dietary preferences. The results of our morphometric analyses support this hypothesis. We found embryonic plagiopatagial shape to be preserved in over a dozen bats with diverse adult plagiopatagial shapes at St 15 and St 16. Not until St 17, after the plagiopatagium and limb have merged, does plagiopatagial shape begin to become more distinct in these bats. At this stage, plant-visiting bats that forage in clutter and/or narrow spaces tend to cluster along PC1, with the edge-space foragers *Noctilio* and *Saccopteryx* defining the extreme negative values along this axis. *Hipposideros*, an insectivorous taxon that tends to forage in edge-space, clusters with the phyllostomids on PC1 but not PC2. This observation is consistent with PC1 being more associated with foraging style and PC2 with diet. However, the distribution of bats in the St 17 morphospace remains distinct from what would be expected for adult bats. For example, open-space-foraging bats like *Molossus* cluster more closely with clutter-foraging bats than with *Noctilio* and *Saccopteryx* at St 17 (Fig. 1D). This suggests that the plagiopatagial morphology observed at St 17 likely only captures the beginning of shape diversification and that this process continues at later developmental stages. Taken together, our findings are consistent with foraging/diet-relevant plagiopatagial shapes arising through differential growth after merging of the plagiopatagia and limb rather than through changes in initial patterning. It is quite possible that membrane shape variation resulting from selection pressures other than foraging/diet (e.g., roosting behaviors, reproductive demands, predator avoidance) also arises after the plagiopatagia and limb have merged. However, as we did not explicitly test this hypothesis in this study, it will need to be assessed through future research. Furthermore, while membrane shape is likely influenced by tissue interactions with the supporting skeletal structures of the limbs and tail and by differential growth rates within the developing plagiopatagium, our study did not explicitly examine the specific developmental processes (e.g., differential growth, gene expression changes) driving divergences in plagiopatagial shape; future research is needed to pursue this interesting topic.

While wing membranes are necessary for powered flight in bats, membranes that function as airfoils are not restricted to the clade. Sixty-five species of living mammals use plagio- and uro- patagia to glide, i.e., achieve “unpowered” flight [90]. These species are spread throughout the mammalian family tree and include representatives from 6 extant families and both the eutherian and marsupial lineages. Multiple fossil mammals and mammaliaformes also have extensive patagia and, as a result, are hypothesized to have been capable of gliding [91–93]. Furthermore, pterosaurs’ flight capabilities were largely dependent on the presence of a plagiopatagium. Unfortunately, little is known about patagial development outside of the bat clade. Given the anatomical similarities between the patagia of bats and extant gliders, it is possible that differential regulation of epithelial development could play a role in convergent patagial evolution across species. Further research is needed to explore this hypothesis.

## Conclusions

To conclude, by providing morphological, molecular, and cellular insights into the development of a novel structure and its subsequent morphological diversification, this study advances our understanding of how morphological novelties emerge and might diversify during the colonization of various ecological niches [94, 95]. Results of this study also provide a strong foundation for the years of additional morphological, molecular, and cellular research needed to fully characterize the developmental basis of the initial outgrowth, merging, and subsequent morphological diversification of the bat plagiopatagium.

## Methods

### Animal collection

For histology, in situ hybridization, scanning electron microscopy (SEM), and RNA-Seq analysis, bat tissues were collected during approved fieldwork in the Dominican Republic, Puerto Rico, and Trinidad. Additional *Carollia perspicillata* samples for IF and further histological analyses were acquired from the Zoological Society of London (ZLS) during a scheduled cull of their colony. Except for tissues to be used for RNA-Seq and SEM, all new tissues collected for this project were fixed overnight at 4 °C in 4% methanol-free formaldehyde (PFA) in 1 × phospho-buffered saline (PBS) before being dehydrated through a series of graded alcohols and stored at − 20 °C until use. Tissues for RNA-Seq analysis were immersed overnight at 4 °C in RNAlater® (Ambion) and then stored in RNAlater® at − 20 °C until use. Tissues for SEM were fixed in 2% formaldehyde and 2.5% glutaraldehyde, washed in PBS, then stained with 1% osmium tetroxide before being brought stepwise into 100% ethanol for storage at − 20 °C until use [96]. All bat embryos were staged following Cretekos et al. [60]. Further details for the bat samples used in this study are provided in Additional File 2: Table S2 (samples used for histological, gene expression, and SEM analyses) and 3 (samples used for gross anatomical and morphometric analyses).

### Geometric morphometrics

Embryos obtained during approved fieldwork in the Dominican Republic, Puerto Rico, and Trinidad and from preserved museum specimens in the collections of the American Museum for Natural History (AMNH; Additional File 2: Table S3) were used for geometric morphometric analysis. Embryos housed in the AMNH were typically extracted from pregnant females that had been captured in the wild by previous investigators, fixed in formalin and/or 70% alcohol, and kept in 70% ethanol at room temperature for a number of years. Ninety-three individual embryos between St 15 and 17, from 37 species, were imaged in a standardized orientation against a millimeter scale. These equated to 21 individuals for St 15, 38 individuals for St 16, and 32 individuals for St 17 (see Additional File 2: Table S3 for details). A literature review was carried out to determine the diet, and foraging habitat for each species (Additional File 2: Table S1). Two-dimensional coordinates were obtained from 13 landmark points using FIJI (ImageJ 1.47v) [97, 98]. These landmarks (Fig. 4B) are defined as:

1. Superficial location of distal end of ulna
2. Most distal connection of the patagium to the forelimb along the fifth digit
3. Apex of anterior plagiopatagium curve
4. Midpoint of slope between 3 and 5
5. Point where anterior curve levels off and goes parallel with body wall
6. Point where parallel ceases and begins posterior arc toward hindlimb
7. Most distal connection along hindlimb
8. Most proximal connection to hindlimb
9. Posterior curve from hindlimb to body wall
10. Anterior curve from forelimb to body wall
11. Most proximal connection to forelimb
12. Superficial location of humerus-radius joint
13. Midpoint of forelimb curve

Procrustes fit was carried out using MorphoJ [99], and covariance matrices were generated. Variances were compared using principal component analysis (PCA). Non-parametric Kruskal–Wallis tests were used to assess whether the medians of the PCs for foraging activity groups (e.g., edge, open) statistically differed within Stages 15, 16, and 17.

### Tissue processing and histological staining

For histological, IF, and TUNEL staining (the latter two described in more detail below), alcohol-dehydrated samples were wax embedded by clearing with Histoclear II followed by infiltration with paraffin wax at 60 °C. Wax-embedded samples were then microtome sectioned at 8-µm thickness and mounted in parallel series on charged slides. The resulting slides were stained with H&E for histological examinations using standard techniques. Slides were then imaged on a Nikon Eclipse 50i microscope. Due to limitations in the availability of tissues across all species, H&E staining was carried out on a single individual per stage for each species.

### RNA sequencing and data analysis

For RNA-Seq, total RNA was isolated from RNAlater®-preserved tissue by homogenization followed using an EZNA Total RNA kit (Omega BioTek). RNA-Seq Libraries were then constructed using an Illumina TruSeq Stranded mRNA kit. High-throughput sequencing was conducted on an Illumina HiSeq 4000 housed at the W.M. Keck Center for Comparative and Functional Genomics at the University of Illinois (UIUC). RNA-Seq analysis was conducted on the UIUC Web-based Galaxy platform (galaxy.illinois.edu) using the Tuxedo protocol [100]. Heart, uropatagial, plagiopatagial, and forelimb tissues were sequenced for three individuals for Stages 14 and 16 for two species (4 tissues per individual, 12 individuals total):

#### *Pteronotus quadridens* and *Erophylla sezekorni*

Sequencing resulted in over 1 million transcripts for each species. These were mapped to the *Miniopterus natalensis* genome [40], which collapsed on to around 800,000 genes per species. To prevent unwanted noise, genes with low expression levels were then filtered out using a criterion of greater than 5 counts presenting in at least 3 samples, resulting in 147,478 and 185,841 genes for *Erophylla* and *Pteronotus*, respectively. Multidimensional clustering analysis was carried out on the 500 most variable genes after filtering to determine the effect of the tissue and stage. A pairwise comparison of each stage and tissue was carried out at 1-way ANOVA with an FDR of *p* < 0.01.

### Wholemount in situ hybridization chain reaction (HCR)

Differential expression of select RNA-Seq genes, specifically *Ripk4*, *Klf4*, and *Tmeff2*, was confirmed by fluorescence in situ hybridization (FISH). FISH was performed using probes designed against conserved regions of each gene in each species by Molecular Instruments on wholemount *Pteronotus quadridens* and *Erophylls sezekorni* tissues from St 14 and St 16. Briefly, samples were rehydrated to 1 × PBS, treated with proteinase K, and post-fixed in 4% PFA. Samples were then treated with 1% H_2_O_2_, washed in 1 × PBS, and equilibrated with hybridization solution. Samples were then hybridized with probe for 12–14 h at 37 °C. Extra probe was then washed off through multiple buffer washes at 37 °C. The probe signal was amplified through incubation with fluorescently labelled hairpin stands for 12–16 h. Excess hairpin reagent was removed through SSCT washes. Resulting samples were cleared in Scale A2 solution and imaged using a Zeiss LSM 700 confocal microscope housed at UIUC. FISH was repeated in two or three individuals, depending on tissue availability, for each stage and species.

### IF and TUNEL assays

IF and TUNEL assays were performed only in *Carollia perspicillata* embryos obtained from the Zoological Society of London, described above. These samples were used for this purpose because they were collected, fixed, and stored in optimal laboratory conditions. As such they were the best tissues available for these analyses; many field-collected samples demonstrated unacceptable levels of background and autofluorescence or had varying levels of staining across slides or sections (data not shown). *Carollia perspicillata* has the added advantage of being readily bred in a non-zoo/research setting. This has permitted its embryonic development to be examined at carefully timed stages from fertilization until parturition. This presently is not possible with any other bat species [34, 39, 51, 60, 82].

For IF, slides were rehydrated through a graded ethanol series to 1 × PBS. Heat-induced antigen retrieval was carried out by microwaving samples for 15 min in preheated 0.1 M Sodium citrate pH6 buffer. Slides were then blocked in 1% bovine serum albumin, 0.1% cold water fish skin gelatin, and 0.1% triton-X for 1 h. Sections were then treated overnight at 4 °C with primary antibodies. The following primary antibodies were used: Rabbit anti-E-Cadherin (Abcam ab76319, 1:200 dilution), mouse anti-cytokeratin 17 (Santa Cruz sc-393002, 1:200 dilution), rabbit anti-pan cytokeratin (Dako Z0622, 1:1000 dilution), rabbit anti-PCNA (Abcam ab193965 1:1000 dilution), rabbit anti-cleaved Caspase 3 (Cell Signaling #9661, 1:400 dilution), and rabbit anti-P63 (Abcam ab124762, 1:200 dilution). Following repeated PBS washes, appropriate secondary antibodies were added. The following secondary antibodies were used at 1:300 dilution: Alexa 488 conjugated goat anti-Rabbit (Invitrogen A11008), Alexa488 conjugated goat anti-Mouse (Invitrogen A11001), and Alexa568 conjugated Donkey anti-Rabbit (Invitrogen A10042). Secondary antibodies were added in blocking buffer for 1 h at room temperature in the dark. Secondary antibody was then washed off using 1 × PBS and slides mounted with Fluoroshield mounting medium containing DAPI (Abcam). TUNEL assays for apoptotic cell death were carried out using the ApopTag Fluorescein In Situ Apoptosis Detection Kit (Merk). IF and TUNEL-stained sections were imaged on a Zeiss Apotome Fluorescence microscope housed at the Centre for Craniofacial and Regenerative Biology, King’s College London. Each assay was repeated in tissues from two separate individuals for each examined developmental stage.

### SEM

*Carollia perspicillata* embryos collected during fieldwork in Trinidad were used for SEM. To prepare for SEM, embryos were dried in a critical point drier and then sputter coated with gold palladium. Morphology was then visualized using an Environmental Scanning Electron Microscope (Phillips X130 ESEM-FEG manufactured by FEI Company) housed in the Imaging Technology Group of the Beckman Institute (UIUC).

### Cell counts

H & E images covering the entire plagiopatagium regions of *Artibeus jamaicensis*,* Carollia perspicillata*,* Erophlla sezekorni*,* Glossophaga soricina*,* Macrotus waterhousii*,* Molossus molossus*,* Pteronotus quadridens*, and *Sturnira erythromos* embryos at St 14, St 15, St 16, and St 17 were imported into FIJI software. Due to tissue availability, a maximum of one individual was used per stage for each species. The Cell Count plug-in was used to count the number of cell layers in the epidermal epithelium. The mean number of cells was counted for each image from a minimum of 10 basal–apical cell stacks. The mean was then calculated across three to five sections and plotted using Prism 9 (Graphpad).

## Supplementary Information

**Additional file 1: Fig S1.** Gross morphology of 16 species of bats between Stage 14 and 16. **Fig S2.** Plagiopatagial HCR in situ hybridization for Ripk4, Tmeff2, and Klf4 for Pteronotus and Erophylla plagiopatagia. **Fig S3.** Principal component plots of geometric morphometric analysis of the developing plagiopatagium. **Fig S4.** Hematoxylin and eosin-stained sections through the plagiopatagium of eight species of bats between Stage 14 and Stage 17.

**Additional file 2: Table S1.** Diet and foraging habitat information of species used in morphometric analysis [102–130]. **Table S2.** Fieldwork and Zoo Samples used for histological, SEM, and gene expression analysis. Table S3. Source of embryo samples used for gross anatomical and morphometric analysis.

## Data Availability

RNA-Seq and associated data from these experiments have been deposited in the GEO database of NCBI under entry GSE224088 and are publicly available [101].

## Abbreviations

AMNH: American Museum for Natural History
evo-devo: Evolutionary developmental biology
FISH: Fluorescence in situ hybridization
H & E: Hematoxylin and eosin
IF: Immunofluorescence assay
MDS: Glimma multidimensional scaling
PBS: Phospho-buffered saline
PC: Principal component
PCA: Principal component analysis
PFA: 4% Paraformaldehyde
SEM: Scanning electron microscopy
St: Stage
ZLS: Zoological Society of London

## References

[1] Wagner GP (2014). Homology, genes, and evolutionary innovation.

[2] Pfennig DW, Wund MA, Snell-Rood EC, Cruickshank T, Schlichting CD, Moczek AP (2010). Phenotypic plasticity’s impacts on diversification and speciation. Trends Ecol Evol.

[3] Klingenberg CP (2019). Phenotypic plasticity, developmental instability, and robustness: the concepts and how they are connected. Front Ecol Evol.

[4] Dworkin I (2005). A study of canalization and developmental stability in the sternopleural bristle system of Drosophila melanogaster. Evolution.

[5] Wagner GP, Booth G, Bagheri-Chaichian H (1997). A population genetic theory of canalization. Evolution.

[6] Orr HA (1998). The population genetics of adaptation: the distribution of factors fixed during adaptive evolution. Evolution.

[7] Moczek AP, Sultan S, Foster S, Ledón-Rettig C, Dworkin I, Nijhout HF, Abouheif E, Pfennig DW (2011). The role of developmental plasticity in evolutionary innovation. Proc R Soc Lond B Biol Sci.

[8] Oliver JC, Tong XL, Gall LF, Piel WH, Monteiro A (2012). A single origin for nymphalid butterfly eyespots followed by widespread loss of associated gene expression. PLoS Genet.

[9] Keys DN, Lewis DL, Selegue JE, Pearson BJ, Goodrich L, Johnson RL, Gates J, Scott MP, Carroll SB (1999). Recruitment of a hedgehog regulatory circuit in butterfly eyespot evolution. Science.

[10] Carroll SB, Gates J, Keys DN, Paddock SW, Panganiban GEF, Selegue JE, Williams JA (1994). Pattern formation and eyespot determination in butterfly wings. Science.

[11] Shirai LT, Saenko SV, Keller RA, Jeránimo MA, Brakefield PM, Descimon H, Wahlberg N, Beldade P (2012). Evolutionary history of the recruitment of conserved developmental genes in association to the formation and diversification of a novel trait. BMC Evol Biol.

[12] Held LI (2013). Rethinking butterfly eyespots. Evol Biol.

[13] Hu Y, Linz DM, Moczek AP (2019). Beetle horns evolved from wing serial homologs. Science.

[14] Averof M, Patel NH (1997). Crustacean appendage evolution associated with changes in Hox gene expression. Nature.

[15] Fritsch M, Richter S (2017). Unexpected UBX expression in the maxilliped of the mystacocarid crustacean Derocheilocharis remanei - evidence for a different way of making a maxilliped?. Dev Genes Evol.

[16] Tokita M, Kiyoshi T, Armstrong KN (2007). Evolution of craniofacial novelty in parrots through developmental modularity and heterochrony. Evol Dev.

[17] Gunnell GF, Simmons NB (2005). Fossil evidence and the origin of bats. J Mamm Evol.

[18] Smith T, Habersetzer J, Simmons NB, Gunnell GF (2012). Systematics and paleobiogeography of early bats. Evolutionary History of Bats: Fossils, Molecules and Morphology.

[19] Simmons NB, Seymour KL, Habersetzer J, Gunnell GF (2008). Primitive early Eocene bat from Wyoming and the evolution of flight and echolocation. Nature.

[20] Sears KE, Behringer RR, Rasweiler JJ, Niswander LA (2006). Development of bat flight: morphologic and molecular evolution of bat wing digits. Proc Natl Acad Sci U S A.

[21] Voigt CC, Frick WF, Holderied MW, Holland R, Kerth G, Mello MAR, Plowright RK, Swartz S, Yovel Y (2017). Principles and patterns of bat movements: from aerodynamics to ecology. Q Rev Biol.

[22] Norberg UM, Rayner JM (1987). Ecological morphology and flight in bats (Mammalia; Chiroptera): wing adaptations, flight perforance, foraging strategy, and echolocation. Philos Trans R Soc Lond B Biol Sci.

[23] Swartz SM, Iriarte-Diaz J, Riskin DK, Song A, Tian X, Willis DJ, Breuer S (2007). Wing structure and the aerodynamic basis of flight in bats. Collection of Technical Papers - 45th AIAA Aerospace Sciences Meeting.

[24] Swartz SM, Konow N (2014). Advances in the study of bat flight: the wing and the wind. Can J Zool.

[25] Swartz SM, Iriarte-Díaz J, Riskin DK, Breuer KS, Gunnell GF, Simmons NB (2012). A bird? A plane? No, it’s a bat: an introduction to the biomechanics of bat flight. Evolutionary History of Bats.

[26] Riskin DK, Racey PA (2010). How do sucker-footed bats hold on, and why do they roost head-up?. Biol J Linn Soc Lond.

[27] Csorba G, Görföl T, Wiantoro S, Kingston T, Bates PJJ, Huang JCC (2015). Thumb-pads up-a new species of thick-thumbed bat from Sumatra (Chiroptera: Vespertilionidae: Glischropus). Zootaxa.

[28] Kock D, Kovac D (2000). Eudiscopus denticulus (Osgood 1932) in Thailand with notes on its roost (Chiroptera: Vespertilionidae). Z Saugetierkd.

[29] Kohles JE, Page RA, Dechmann DKN, O’Mara MT (2018). Rapid behavioral changes during early development in Peters’ tent-making bat (Uroderma bilobatum). PLoS ONE.

[30] Rodrigues-Duran A, Kunz TH (1992). Pteronotus quadridens. Mamm Species.

[31] Norberg UM (2012). Vertebrate flight: Mechanics, physiology, morphology, ecology, and evolution.

[32] Kunz T, Fenton M (2005). Bat ecology.

[33] Mason MK, Hockman D, Curry L, Cunningham TJ, Duester G, Logan M, Jacobs D, Illing N (2015). Retinoic acid-independent expression of Meis2 during autopod patterning in the developing bat and mouse limb. EvoDevo.

[34] Hockman D, Cretekos CJ, Mason MK, Behringer RR, Jacobs DS, Illing N (2008). A second wave of sonic hedgehog expression during the development of the bat limb. Proc Natl Acad Sci U S A.

[35] Maier JA, Rivas-Astroza M, Deng J, Dowling A, Oboikovitz P, Cao X, Behringer R, Cretekos C, Rasweiler JJ, Zhong S, Sears KE (2017). Transcriptomic insights into the genetic basis of mammalian limb diversity. BMC Evol Biol.

[36] Dai M, Wang Y, Fang L, Irwin DM, Zhu T, Zhang J, Zhang S, Wang Z (2014). Differential expression of Meis2, Mab21l2 and Tbx3 during limb development associated with diversification of limb morphology in mammals. PLoS ONE.

[37] Cretekos CJ, Wang Y, Green ED, Martin JF, Rasweiler JJ, Behringer RR (2008). Regulatory divergence modifies limb length between mammals. Genes Dev.

[38] Wang Z, Dai M, Wang Y, Cooper KL, Zhu T, Dong D, Zhang J, Zhang S (2014). Unique expression patterns of multiple key genes associated with the evolution of mammalian flight. Proc R Soc Lond B Biol Sci.

[39] Chen CH, Cretekos CJ, Rasweiler JJ, Behringer RR (2005). Hoxd13 expression in the developing limbs of the short-tailed fruit bat. Carollia perspicillata Evol Dev.

[40] Eckalbar WL, Schlebusch SA, Mason MK, Gill Z, Parker A, Booker BM, Nishizaki S, Muswanba-Nday C, Terhune E, Nevonen KA, Makki N, Friedrich T, VanderMeer JE, Pollard KS, Carbone L, Wall JD, Illing N, Ahituv N (2016). Transcriptomic and epigenomic characterization of the developing bat wing. Nat Genet.

[41] Booker BM, Friedrich T, Mason MK, VanderMeer JE, Zhao J, Eckalbar WL, Logan M, Illing N, Pollars KS, Ahituv N (2016). Bat accelerated regions identify a bat forelimb specific enhancer in the HoxD locus. PLoS Genet.

[42] Cooper LN, Sears KE (2013). How to grow a bat wing. Bat Evolution, Ecology, and Conservation.

[43] Howenstine AO, Sadier A, Anthwal N, Lau CL, Sears KE (2021). Non-model systems in mammalian forelimb evo-devo. Curr Opin Genet Dev.

[44] López-Aguirre C, Hand SJ, Koyabu D, Son NT, Wilson LAB (2019). Postcranial heterochrony, modularity, integration and disparity in the prenatal ossification in bats (Chiroptera). BMC Evol Biol.

[45] López-Aguirre C, Hand SJ, Koyabu D, Son NT, Wilson LAB (2019). Prenatal allometric trajectories and the developmental basis of postcranial phenotypic diversity in bats (Chiroptera). J Exp Zool B Mol Dev Evol.

[46] Ray R, Capecchi M (2008). An examination of the Chiropteran HoxD locus from an evolutionary perspective. Evol Dev.

[47] Petit F, Sears KE, Ahituv N (2017). Limb development: a paradigm of gene regulation. Nat Rev Genet.

[48] Farnum CE, Tinsley M, Hermanson JW (2007). Forelimb versus hindlimb skeletal development in the big brown bat, Eptesicus fuscus: functional divergence is reflected in chondrocytic performance in autopodial growth plates. Cells Tissues Organs.

[49] Adams RA (2007). Morphogenesis in bat wings: linking development, evolution, and ecology. Cells Tissues Organs.

[50] Merino R, Rodriguez-Leon J, Macias D, Gañan Y, Economides AN, Hurle JM (1999). The BMP antagonist Gremlin regulates outgrowth, chondrogenesis and programmed cell death in the developing limb. Development.

[51] Weatherbee SD, Behringer RR, Rasweiler JJ, Niswander LA (2006). Interdigital webbing retention in bat wings illustrates genetic changes underlying amniote limb diversification. Proc Natl Acad Sci U S A.

[52] Sadier A, Urban DJ, Anthwal N, Howenstine AO, Sinha I, Sears KE (2020). Making a bat: the developmental basis of bat evolution. Genet Mol Biol.

[53] Simmons NB, Cirranello AL (2002). Bat species of the world: a taxonomic and geographic database.

[54] Wang Z, Han N, Racey PA, Ru B, He G (2010). A comparative study of prenatal development in Miniopterus schreibersii fuliginosus, Hipposideros armiger and H. pratti. BMC Dev Biol.

[55] Usui K, Tokita M (2019). Normal embryonic development of the greater horseshoe bat *Rhinolophus*
*ferrumequinum*, with special reference to nose leaf formation. J Morphol.

[56] Khannoon ER, Usui K, Tokita M (2020). Embryonic development of the Egyptian fruit bat Rousettus aegyptiacus (Mammalia: Chiroptera: Pteropodidae). Acta Chiropt.

[57] Giannini N, Goswami A, Sánchez-Villagra MR (2006). Development of integumentary structures in Rousettus amplexicaudatus (Mammalia: Chiroptera: Pteropodidae) during late-embryonic and fetal stages. J Mammal.

[58] Lawrence MA (1991). Biological observations on a collection of New Guinea Syconycteris australis (Chiroptera, Pteropodidae) in the American Museum of Natural History. Am Mus Novit.

[59] Ventura A, Nogueira MR, Peracchi AL, do Nascimento AA, Vieira-Lopes DA, Pinheiro NL (2018). Comparative prenatal development and embryonic staging of neotropical fruit bats (genus Artibeus). Zool Anz.

[60] Cretekos CJ, Weatherbee SD, Chen CH, Badwaik NK, Niswander L, Behringer RR, Rasweiler JJ (2005). Embryonic staging system for the short-tailed fruit bat, Carollia perspicillata, a model organism for the mammalian order Chiroptera, based upon timed pregnancies in captive-bred animals. Dev Dyn.

[61] Hockman D, Mason MK, Jacobs DS, Illing N (2009). The role of early development in mammalian limb diversification: a descriptive comparison of early limb development between the natal long-fingered bat (Miniopterus natalensis) and the mouse (Mus musculus). Dev Dyn.

[62] Wyant KA, Adams RA (2007). Prenatal growth and development in the Angolan free-tailed bat, Mops condylurus (Chiroptera: Molossidae). J Mammal.

[63] Rodríguez FE, Sandoval MT, Álvarez BB, Lombardo DM (2018). Comparative study of prenatal development between *Myotis*
*albescens* (Chiroptera: Vespertilionidae) and *Eumops*
*patagonicus* (Chiroptera: Molossidae): the chorionic vesicle and extraembryonic membranes considerations. Anat Rec.

[64] Paksuz EP, Hayretdağ S, Olgun K (2017). Prenatal development in greater mouse-eared bat, *Myotis*
*myotis* (Borkhausen, 1797) (Chiroptera, Vespertilionidae). Anat Histol Embryol.

[65] Nolte MJ, Hockman D, Cretekos CJ, Behringer RR, Rasweiler JJ (2009). Embryonic staging system for the black mastiff bat, Molossus rufus (Molossidae), correlated with structure-function relationships in the adult. Anat Rec.

[66] Tokita M (2006). Normal embryonic development of the Japanese pipistrelle, Pipistrellus abramus. Zoology (Jena).

[67] Nojiri T, Fukui D, Werneburg I, Saitoh T, Endo H, Koyabu D (2021). Embryonic staging of bats with special reference to *Vespertilio*
*sinensis* and its cochlear development. Dev Dyn.

[68] Tokita M, Abe T, Suzuki K (2012). The developmental basis of bat wing muscle. Nat Commun.

[69] Kalay E, Sezgin O, Chellappa V, Mutlu M, Morsy H, Kayserili H, Kreiger E, Cansu A, Toraman B, Abdalla EM, Aslan Y, Pillai S, Akarsu A (2012). Mutations in RIPK4 cause the autosomal-recessive form of popliteal pterygium syndrome. Am J Hum Genet.

[70] Mitchell K, O’Sullivan J, Missero C, Blair E, Richardson R, Anderson B, Antonini D, Murray JC, Shanske AL, Schutte BC, Romano RA, Sinha S, Bhaskar SS, Black GCM, Dixon J, Dixon MJ (2012). Exome sequence identifies RIPK4 as the Bartsocas-Papas syndrome locus. Am J Hum Genet.

[71] Oberbeck N, Pham VC, Webster JD, Reja R, Huang CS, Zhang Y, Roose-Girma M, Warming S, Li Q, Birnberg A, Wong W, Sandoval W, Komuves LG, Yu K, Dugger DL, Maltzman A, Newton K, Dixit VM (2019). The RIPK4–IRF6 signaling axis safeguards epidermal differentiation and barrier function. Nature.

[72] Holland PM, Willis CR, Kanaly S, Glaccum MB, Warren AS, Charrier K, Murison J, Derry J, Virca G, Bird T, Peschon J (2002). RIP4 is an ankyrin repeat-containing kinase essential for keratinocyte differentiation. Curr Biol.

[73] de Groote P, Tran HT, Fransen M, Tanghe G, Urwyler C, de Craene B, Leurs K, Gilbert B, Van Imschoot G, De Rycke R, Guerin CJ, Holland P, Berx G, Vandenabelle P, Lippens S, Vleminckx K, Declercq W (2015). A novel RIPK4-IRF6 connection is required to prevent epithelial fusions characteristic for popliteal pterygium syndromes. Cell Death Differ.

[74] Richardson RJ, Hammond NL, Coulombe PA, Saloranta C, Nousiainen HO, Salonen R, Berry A, Hanley N, Headon D, Karikoski R, Dixon MJ (2014). Periderm prevents pathological epithelial adhesions during embryogenesis. J Clin Invest.

[75] Munin RL, Fischer E, Gonçalves F (2012). Food habits and dietary overlap in a phyllostomid bat assemblage in the pantanal of Brazil. Acta Chiropt.

[76] Segre JA, Bauer C, Fuchs E (1999). Klf4 is a transcription factor required for establishing the barrier function of the skin. Nat Genet.

[77] Hammond NL, Dixon J, Dixon MJ (2019). Periderm: life-cycle and function during orofacial and epidermal development. Semin Cell Dev Biol.

[78] Lough KJ, Byrd KM, Spitzer DC, Williams SE (2017). Closing the gap: mouse models to study adhesion in secondary palatogenesis. J Dent Res.

[79] Sur I, Rozell B, Jaks V, Bergström Å, Toftgård R (2006). Epidermal and craniofacial defects in mice overexpressing Klf5 in the basal layer of the epidermis. J Cell Sci.

[80] Wang Z, Dong D, Ru B, Young RL, Han N, Guo T, Zhang S (2010). Digital gene expression tag profiling of bat digits provides robust candidates contributing to wing formation. BMC Genomics.

[81] McCulloch DR, Nelson CM, Dixon LJ, Silver DL, Wylie JD, Lindner V, Sasaki T, Cooley MA, Argraves WS, Apte SS (2009). ADAMTS metalloproteases generate active versican fragments that regulate interdigital web regression. Dev Cell.

[82] Cretekos CJ, Deng JM, Green ED, Rasweiler JJ, Behringer RR (2007). Isolation, genomic structure, and developmental expression of Fgf8 in the short-tailed fruit bat, Carollia perspicillata. Int J Dev Biol.

[83] Senoo M, Pinto F, Crum CP, McKeon F (2007). p63 Is Essential for the proliferative potential of stem cells in stratified epithelia. Cell.

[84] Kashgari G, Meinecke L, Gordon W, Ruiz B, Yang J, Ma AL, Xie Y, Ho H, Plikus MV, Nie Q, Jester JV, Anderson B (2020). Epithelial migration and non-adhesive periderm are required for digit separation during mammalian development. Dev Cell.

[85] Fons JM, Mozaffari M, Malik D, Marshall AR, Connor S, Greene NDE, Tucker AS (2020). Epithelial dynamics shed light on the mechanisms underlying ear canal defects. Development.

[86] Orr DJA, Teeling EC, Puechmaille SJ, Finarelli JA (2016). Patterns of orofacial clefting in the facial morphology of bats: a possible naturally occurring model of cleft palate. J Anat.

[87] Usui K, Tokita M (2018). Creating diversity in mammalian facial morphology: a review of potential developmental mechanisms. EvoDevo.

[88] Curtis AA, Arbour JH, Santana SE (2020). Mind the gap: natural cleft palates reduce biting performance in bats. J Exp Biol.

[89] Giannini NP, Simmons NB (2007). The chiropteran premaxilla: a reanalysis of morphological variation and its phylogenetic interpretation. Am Mus Novit.

[90] Jackson S, Schouten P (2019). Gliding mammals of the world.

[91] Meng QJ, Grossnickle DM, Liu D, Zhang YG, Neander AI, Ji Q, Luo ZX (2017). New gliding mammaliaforms from the Jurassic. Nature.

[92] Han G, Mao F, Bi S, Wang Y, Meng J (2017). A Jurassic gliding euharamiyidan mammal with an ear of five auditory bones. Nature.

[93] Meng J, Hu Y, Wang Y, Wang X, Li C (2006). A Mesozoic gliding mammal from northeastern China. Nature.

[94] Muller GB, Wagner GP (1991). Novelty in evolution: restructuring the concept. Annu Rev Ecol Syst.

[95] Moczek AP, Sears KE, Stollewerk A, Wittkopp PJ, Diggle P, Dworkin I, Ledon-Rettig C, Matus DQ, Roth S, Abouheif E, Brown FD, Chiu CH, Cohen CS, De Tomaso AW, Gilbert SF, Hall B, Love AC, Lyons DC, Sanger TJ, Smith J, Specht C, Vallejo-Marin M, Extavour CG (2015). The significance and scope of evolutionary developmental biology: a vision for the 21st century. Evol Dev.

[96] Nagy A, Gertsenstein M, Vintersten K, Behringer RR (2002). Manipulating the mouse embryo: a laboratory manual.

[97] Schindelin J, Rueden CT, Hiner MC, Eliceiri KW (2015). The ImageJ ecosystem: an open platform for biomedical image analysis. Mol Reprod Dev.

[98] Schindelin J, Arganda-Carreras I, Frise E, Kaynig V, Longair M, Pietzsch T, Preibisch S, Rueden C, Saalfeld S, Schmid B, Tinevez JY, White DJ, Hartenstein V, Eliceiri K, Tomancak P, Cardona A (2012). Fiji: an open-source platform for biological-image analysis. Nat Methods.

[99] Klingenberg CP (2011). MorphoJ: an integrated software package for geometric morphometrics. Mol Ecol Resour.

[100] Trapnell C, Roberts A, Goff L, Pertea G, Kim D, Kelley DR, Pimental H, Salzberg S, Runn JL, Pachter L (2012). Differential gene and transcript expression analysis of RNA-seq experiments with TopHat and Cufflinks. Nat Protoc.

[101] Sears KE, Urban DJ, Anthwal N. Insights into the formation and diversification of a novel chiropteran wing membrane from embryonic development. 2023. NCBI GEO https://www.ncbi.nlm.nih.gov/geo/query/acc.cgi?acc=GSE224088. 10.1186/s12915-023-01598-y PMC10161559 37143038

[102] Yancey FD, Goetze JR, Jones C. Saccopteryx bilineata. Mamm Species. 1998;581:1–5.

[103] Ratcliffe JM, Jakobsen L, Kalko EKV, Surlykke A (2011). Frequency alternation and an offbeat rhythm indicate foraging behavior in the echolocating bat, Saccopteryx bilineata. J Comp Physiol A Neuroethol Sens Neural Behav Physiol.

[104] Monadjem A, Soisook P, Thong VD, Kingston T (2016). Family Hipposideridae, Old World leaf-nosed bats. Mammals of Africa: hedgehogs, shrews and bats.

[105] Pavey CR (2021). Comparative echolocation and foraging ecology of horseshoe bats (Rhinolophidae) and Old-World leaf-nosed bats (Hipposideridae). Aust J Zool.

[106] Gonsalves L, Bicknell B, Law B, Webb C, Monamy V (2013). Mosquito consumption by insectivorous bats: does size matter?. PLoS ONE.

[107] Crisol-Martínez E, Ford G, Horgan FG, Brown PH, Wormington KR (2017). Ecology and conservation of insectivorous bats in fragmented areas of macadamia production in eastern Australia. Austral Ecol.

[108] Esbérard CEL, Bergallo HG (2010). Foraging activity of the free-tailed bat Molossus molossus (Chiroptera; Molossidae) in southeastern Brazil. Braz J Biol.

[109] Rolfe AK, Kurta A, Clemans DL (2014). Species-level analysis of diets of two mormoopid bats from Puerto Rico. J Mammal.

[110] Macías S, Mora EC (2003). Variation of echolocation calls of Pteronotus quadridens (Chiroptera: Mormoopidae) in Cuba. J Mammal.

[111] Gonçalves F, Munin R, Costa P, Fischer E (2007). Feeding habits of Noctilio albiventris (Noctilionidae) bats in the Pantanal. Brazil Acta Chiropt.

[112] Kalko EKV, Schnitzler H-U, Kaipf I, Grinnell AD (1998). Echolocation and foraging behavior of the lesser bulldog bat, Noctilio albiventris: Preadaptations for piscivory?. Behav Ecol Sociobiol..

[113] Denzinger A, Schnitzler HU (2013). Bat guilds, a concept to classify the highly diverse foraging and echolocation behaviors of microchiropteran bats. Front Physiol.

[114] Hood CS, Pitocchelli J. Noctilio albiventris. Mamm Species. 1983;216:1–7.

[115] Fahr J (2013). Nycteris arge. Mammals of Africa: hedgehogs, shrews and bats.

[116] Lee TE, Dominguez DJ. Ametrida centurio. Mamm Species. 2000;640:1–4.

[117] Ingala MR, Simmons NB, Wultsch C, Krampis K, Provost KL, Perkins SL (2021). Molecular diet analysis of neotropical bats based on fecal DNA metabarcoding. Ecol Evol.

[118] Swanepoel P, Genoways HH. Brachyphylla cavernarum. Mamm Species. 1983;205:1–6.

[119] Soto-Centeno JA, Kurta A (2006). Diet of two nectarivorous bats, Erophylla sezekorni and Monophyllus redmani (Phyllostomidae), on Puerto Rico. J Mammal.

[120] Sánchez Ó, Wilson DE (2016). Food items of Macrotus waterhousii (Chiroptera: Phyllostomidae) in central Mexico. Therya.

[121] Giannini NP, Kalko EKV (2005). The guild structure of animalivorous leaf-nosed bats of Barro Colorado Island, Panama, revisited. Acta Chiropt.

[122] da Silva AG, Gaona O, Medellín RA (2008). Diet and trophic structure in a community of fruit-eating bats in Lacandon Forest, México. J Mammal.

[123] Molinari J, Soriano PJ. Sturnira bidens. Mamm Species. 1987;276:1–4.

[124] Sánchez MS, dos Santos DA (2015). Understanding the spatial variations in the diets of two sturnira bats (Chiroptera: Phyllostomidae) in Argentina. J Mammal.

[125] Amponsah-Mensah K, Cunningham AA, Wood JLN, Ntiamoa-Baidu Y (2019). Seasonal variation in food availability and relative importance of dietary items in the Gambian epauletted fruit bat (Epomophorus gambianus). Ecol Evol.

[126] Law BS (2001). The diet of the common blossom bat (Syconycteris australis) in upland tropical rainforest and the importance of riparian areas. Wildl Res.

[127] Csorba G,  Ujhelyi P (2003). Horseshoe bats of the world (Chiroptera: Rhinolophidae).

[128] Kalko EKV (1995). Insect pursuit, prey capture and echolocation in pipestirelle bats (Microchiroptera). Anim Behav.

[129] Sosa M, de Ascencao A, Sorianao P (1996). Dieta y patrón reproductivo de Rhogeessa minutilla (Chiroptera: Vespertilionidae) en una zona árida de Los Andes de Venezuela Maricela. Rev Biol Trop.

[130] Rydell J, Arita HT, Santos M, Granados J (2002). Acoustic identification of insectivorous bats (Order Chiroptera) of Yucatan. Mexico J Zool.

