# Supplementary material for: Insights into the formation and diversification of a novel chiropteran wing membrane from embryonic development
Source: BMC Biol. 2023 May 4;21:101. doi: 10.1186/s12915-023-01598-y (PMC10161559; doi:10.1186/s12915-023-01598-y)

Additional File 1: Figure S1. Gross morphology of 16 species of bats between Stage 14 and 16. Arrow indicates location of first appearance of plagiopatagium.

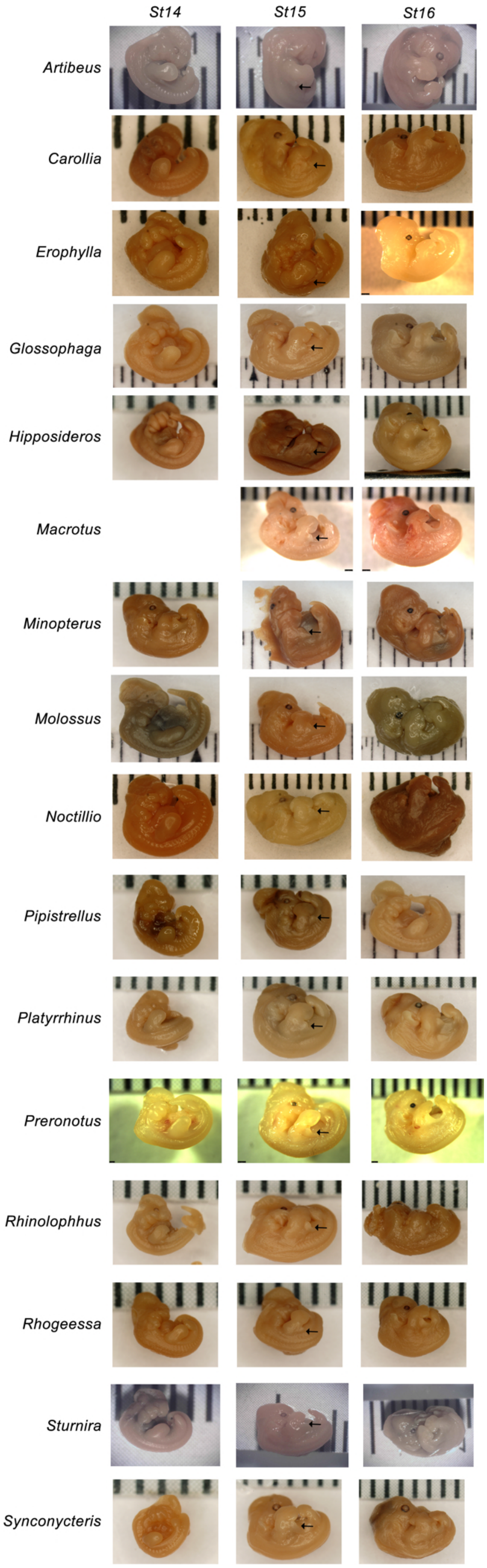

Additional File 1: Figure S2. Plagiopatagial HCR in situ hybridization for Ripk4, Tmeff2, and Klf4 for Pteronotus and Erophylla plagiopatagia at Stage 14 (Pteronotus – A, Erophylla – B) and Stage 16 (Pteronotus – C, Erophylla – D), and merged images for Stage 15 and Stage 17 (Tmeff2 = yellow, Klf4 = magenta, Ripk4 = cyan).

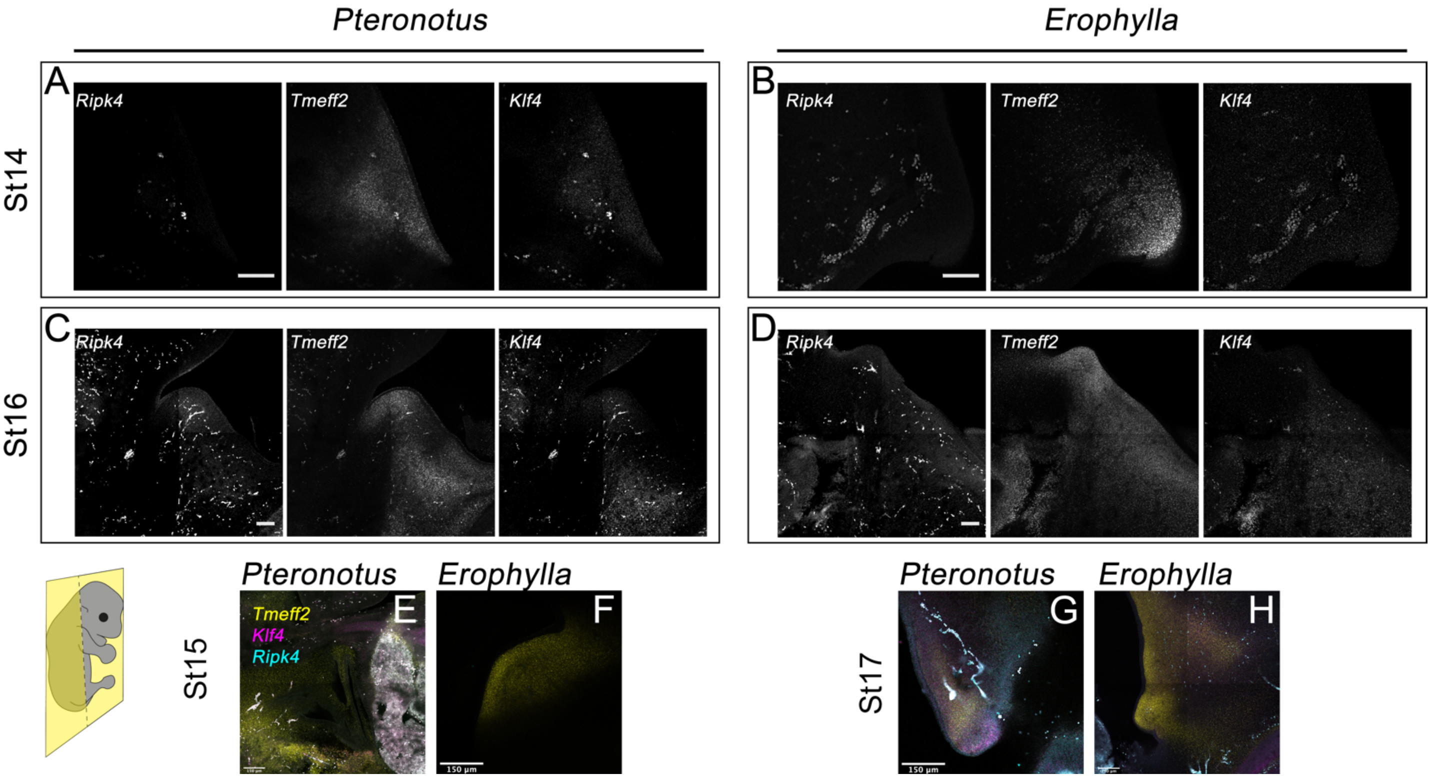

Additional File 1: Figure S3. Principal component plots of geometric morphometric analysis of the developing plagiopatagium, labeled by foraging guild.

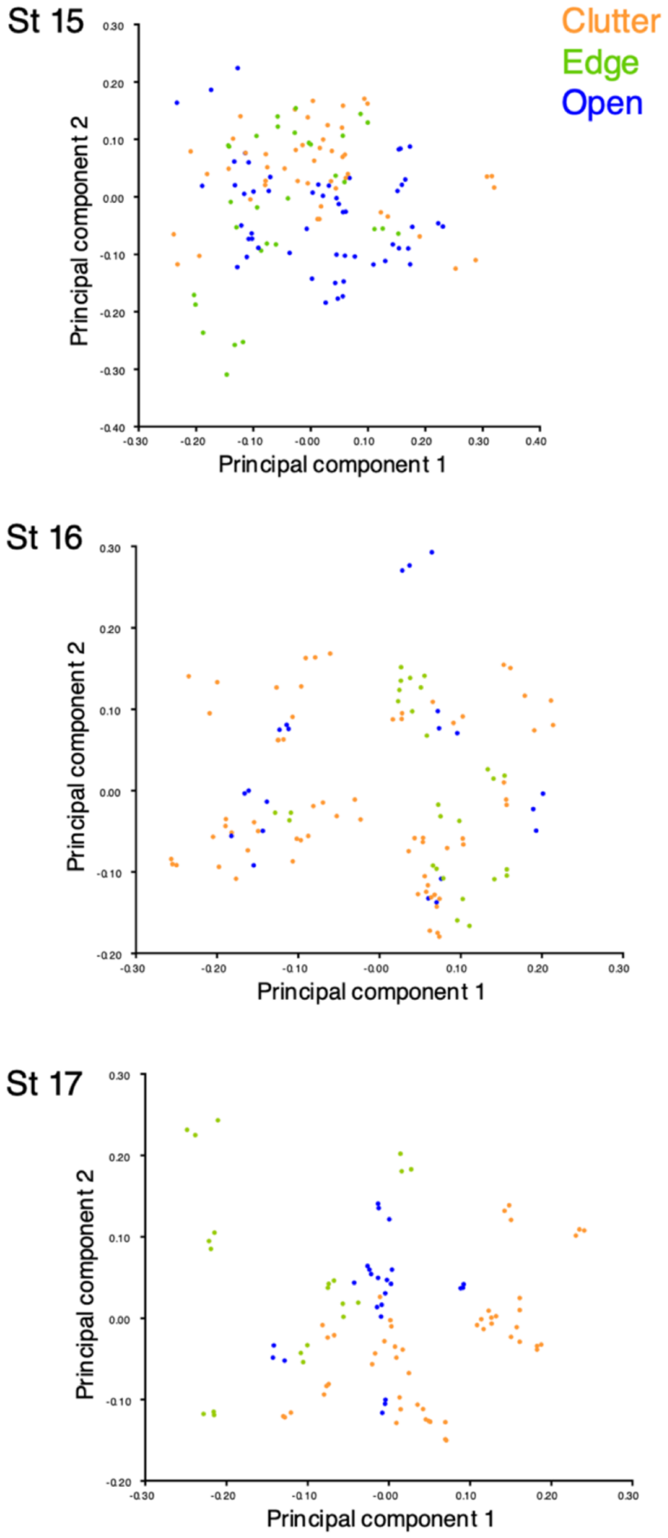

Additional File 1: Figure S4. Hematoxylin and eosin-stained sections through the plagiopatagium of eight species of bats between Stage 14 and Stage 17.

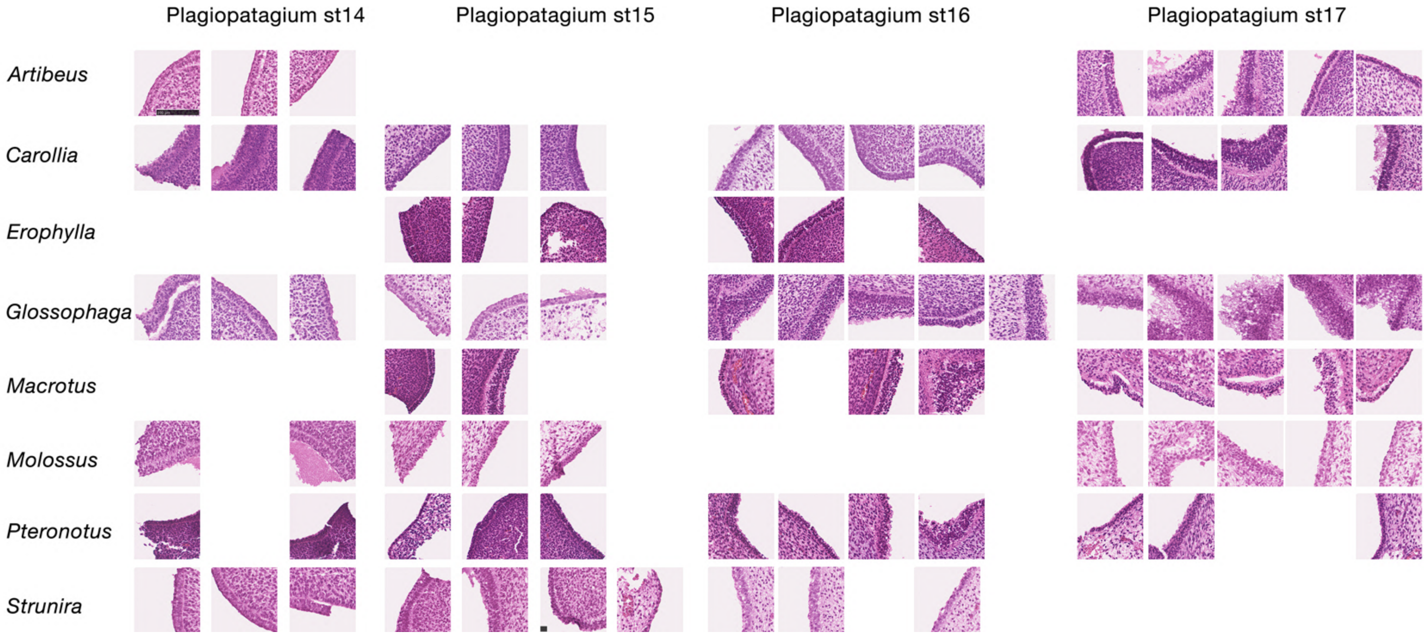

Supplement: Supplementary file 1 — Additional file 1: Fig S1. Gross morphology of 16 species of bats between Stage 14 and 16. Fig S2. Plagiopatagial HCR in situ hybridization for Ripk4, Tmeff2, and Klf4 for Pteronotus and Erophylla plagiopatagia. Fig S3. Principal component plots of geometric morphometric analysis of the developing plagiopatagium. Fig S4. Hematoxylin and eosin-stained sections through the plagiopatagium of eight species of bats between Stage 14 and Stage 17. [file 12915_2023_1598_MOESM1_ESM.pdf]
