# Supplementary material for: Insights into the formation and diversification of a novel chiropteran wing membrane from embryonic development
Source: BMC Biol. 2023 May 4;21:101. doi: 10.1186/s12915-023-01598-y (PMC10161559; doi:10.1186/s12915-023-01598-y)
Supplement: Supplementary file 2 — Additional file 2: Table S1. Diet and foraging habitat information of species used in morphometric analysis [102–130]. Table S2. Fieldwork and Zoo Samples used for histological, SEM, and gene expression analysis. Table S3. Source of embryo samples used for gross anatomical and morphometric analysis. [file 12915_2023_1598_MOESM2_ESM.docx]

Additional File 2: Table S1. Diet and foraging habitat information of species used in morphometric analysis. When multiple closely related species of the same genus were sampled, the species with the most individuals studied is listed.

| Family | Species | Diet | Foraging Habitat |
| --- | --- | --- | --- |
| Emballonuridae | Saccopteryx bilineata | Insects [102] | Edge [103] |
| Hipposideridae | Hipposideros larvatus | Insects [104] | Edge [104, 105] |
| Miniopteridae | Miniopterus australis | Insects [106] | Edge [107] |
| Molossidae | Molossus molossus | Insects [108] | Open space [30] |
| Mormoopidae | Pteronotus quadridens | Insects [109] | Narrow Space/Clutter [110] |
| Noctilionidae | Noctilio albiventris | Insects & rarely small fish [111, 112] | Over water / Edge [113, 114] |
| Nycteridae | Nycteris arge, Nycteris hispida | Insects [115] | Open [115] |
| Phyllostomidae | Ametrida centurio | Fruit [116] | Narrow Space/Clutter [113] |
| Phyllostomidae | Artibeus jamaicensis | Fruit, insects, pollen, leaves [81, 117] | Narrow Space/Clutter [113] |
| Phyllostomidae | Brachyphylla cavernarum | Fruit, pollen, nectar, insects [118] | Narrow Space/Clutter [113] |
| Phyllostomidae | Carollia perspicillata | Fruit, nectar, pollen, insects, leaves [81, 117] | Narrow Space/Clutter [113] |
| Phyllostomidae | Erophylla sezekorni | Fruit, nectar, pollen, insects [119] | Narrow Space/Clutter [113] |
| Phyllostomidae | Glossophaga soricina | Nectar, pollen, fruit, floral parts, insects [81, 117] | Narrow Space/Clutter [113] |
| Phyllostomidae | Lophostoma silvicolum | Fruit, pollen, insects, leaves [81] | Narrow Space/Clutter [113] |
| Phyllostomidae | Macrotus waterhousii | Insects, fruit [120] | Narrow Space/Clutter [120] |
| Phyllostomidae | Monophyllus redmani | Fruit, nectar, pollen, insects [119] | Narrow Space/Clutter [113] |
| Phyllostomidae | Phyllostomus discolor | Fruit, pollen, insects [81, 121] | Narrow Space/Clutter [113] |
| Phyllostomidae | Platyrrhinus helleri | Fruit, insects [122] | Narrow Space/Clutter [113] |
| Phyllostomidae | Sturnira erythromos, Sturnira bidens | Fruit [123, 124] | Narrow Space/Clutter [113] |
| Phyllostomidae | Tonatia saurophila | Insects [121] | Narrow Space/Clutter [113] |
| Phyllostomidae | Trachops cirrhosus | Insects, plants [117, 121] | Narrow Space/Clutter [113] |
| Pteropodidae | Epomophorus gambianus | Fruit, nectar, leaves [125] | Narrow Space/Clutter [113] |
| Pteropodidae | Synconycteris australis | Nectar, pollen, fruit [126] | Narrow Space/Clutter [113] |
| Rhinolophidae | Rhinolophus lepidus, Rhinolophus pearsonii | Insects [127] | Open space [127] |
| *Vespertilionidae* | *Pipistrellus papuanus* | Insects [128] (refers to other *Pipisrellus* species) | Edge [128] (refers to other *Pipisrellus* species) |
| *Vespertilionidae* | *Rhogeessa aeneus, Rhogeessa minutilla* | Insects [117, 129] | Open Space [130] |

Additional File 2: Table S2 - Fieldwork and Zoo Samples used for histological, SEM, and gene expression analysis.

| Species | Carnegie Stage | Sample ID | Collection | Origin | Geographical Origin | Year Collected | Experimental Use |
| --- | --- | --- | --- | --- | --- | --- | --- |
| Erophylla sezekorni | 14 | 2016-026 | UCLA | Fieldwork | Puerto Rico | 2016 | RNA-Sequencing |
| Erophylla sezekorni | 14 | 2016-037 | UCLA | Fieldwork | Puerto Rico | 2016 | RNA-Sequencing |
| Erophylla sezekorni | 14 | 2017-013 | UCLA | Fieldwork | Puerto Rico | 2017 | RNA-Sequencing |
| Pteronotus quadridens | 14 | 2016-119 | UCLA | Fieldwork | Puerto Rico | 2016 | RNA-Sequencing |
| Pteronotus quadridens | 14 | 2017-046 | UCLA | Fieldwork | Puerto Rico | 2017 | RNA-Sequencing |
| Pteronotus quadridens | 14 | 2017-059 | UCLA | Fieldwork | Puerto Rico | 2017 | RNA-Sequencing |
| Erophylla sezekorni | 16 | 2016-030 | UCLA | Fieldwork | Puerto Rico | 2016 | RNA-Sequencing |
| Erophylla sezekorni | 16 | 2015-079 | UCLA | Fieldwork | Puerto Rico | 2015 | RNA-Sequencing |
| Erophylla sezekorni | 16 | 2017-009 | UCLA | Fieldwork | Puerto Rico | 2017 | RNA-Sequencing |
| Pteronotus quadridens | 16 | 2016-101 | UCLA | Fieldwork | Puerto Rico | 2016 | RNA-Sequencing |
| Pteronotus quadridens | 16 | 2016-105 | UCLA | Fieldwork | Puerto Rico | 2016 | RNA-Sequencing |
| Pteronotus quadridens | 16 | 2016-121 | UCLA | Fieldwork | Puerto Rico | 2016 | RNA-Sequencing |
| Pteronotus quadridens | 16 | 2017-048 | UCLA | Fieldwork | Puerto Rico | 2017 | HCR In Situ Hybridization |
| Pteronotus quadridens | 16 | 2017-049 | UCLA | Fieldwork | Puerto Rico | 2017 | HCR In Situ Hybridization |
| Pteronotus quadridens | 15 | 2017-055 | UCLA | Fieldwork | Puerto Rico | 2017 | HCR In Situ Hybridization |
| Pteronotus quadridens | 15 | 2017-056 | UCLA | Fieldwork | Puerto Rico | 2017 | HCR In Situ Hybridization |
| Pteronotus quadridens | 14 | 2017-067 | UCLA | Fieldwork | Puerto Rico | 2017 | HCR In Situ Hybridization |
| Pteronotus quadridens | 14 | 2017-068 | UCLA | Fieldwork | Puerto Rico | 2017 | HCR In Situ Hybridization |
| Pteronotus quadridens | 17 | 2016-117 | UCLA | Fieldwork | Puerto Rico | 2016 | HCR In Situ Hybridization |
| Pteronotus quadridens | 18 | 2016-120 | UCLA | Fieldwork | Puerto Rico | 2016 | HCR In Situ Hybridization |
| Pteronotus quadridens | 18 | 2016-118 | UCLA | Fieldwork | Puerto Rico | 2016 | HCR In Situ Hybridization |
| Pteronotus quadridens | 18 | 2016-138 | UCLA | Fieldwork | Puerto Rico | 2016 | HCR In Situ Hybridization |
| Erophylla sezekorni | 14 | 2015-020 | UCLA | Fieldwork | Puerto Rico | 2015 | HCR In Situ Hybridization |
| Erophylla sezekorni | 14 | 2015-021 | UCLA | Fieldwork | Puerto Rico | 2015 | HCR In Situ Hybridization |
| Erophylla sezekorni | 15 | 2015-012 | UCLA | Fieldwork | Puerto Rico | 2015 | HCR In Situ Hybridization |
| Erophylla sezekorni | 16 | 2015-013 | UCLA | Fieldwork | Puerto Rico | 2015 | HCR In Situ Hybridization |
| Erophylla sezekorni | 16 | 2015-023 | UCLA | Fieldwork | Puerto Rico | 2015 | HCR In Situ Hybridization |
| Erophylla sezekorni | 17 | 2015-024 | UCLA | Fieldwork | Puerto Rico | 2015 | HCR In Situ Hybridization |
| Erophylla sezekorni | 15 | 2015-078 | UCLA | Fieldwork | Puerto Rico | 2015 | HCR In Situ Hybridization |
| Carollia perspicillata | 14 | N/A | University of Texas | Fieldwork | Trinidad | 2005 | Scanning Electron Microscopy |
| Carollia perspicillata | 15 | N/A | University of Texas | Fieldwork | Trinidad | 2005 | Scanning Electron Microscopy |
| Carollia perspicillata | 16 | N/A | University of Texas | Fieldwork | Trinidad | 2005 | Scanning Electron Microscopy |
| Carollia perspicillata | 17 | N/A | University of Texas | Fieldwork | Trinidad | 2005 | Scanning Electron Microscopy |
| Carollia perspicillata | 18 | N/A | University of Texas | Fieldwork | Trinidad | 2005 | Scanning Electron Microscopy |
| Carollia perspicillata | 14 | ZLS015 | King's College London | Zoological Society London | Brazil | 2020 | Immunohistochemistry |
| Carollia perspicillata | 15 | ZLS011 | King's College London | Zoological Society London | Brazil | 2020 | Immunohistochemistry |
| Carollia perspicillata | 16 | ZLS001 | King's College London | Zoological Society London | Brazil | 2020 | Immunohistochemistry |
| Carollia perspicillata | 14 | ZLS014 | King's College London | Zoological Society London | Brazil | 2020 | Immunohistochemistry |
| Carollia perspicillata | 15 | ZLS046 | King's College London | Zoological Society London | Brazil | 2020 | Immunohistochemistry |
| Carollia perspicillata | 16 | ZLS004 | King's College London | Zoological Society London | Brazil | 2020 | Immunohistochemistry |

Additional File 2: Table S3. Source of embryo samples used for gross anatomical and morphometric analysis. ND: No Data.

| Species | Carnegie Stage | Sample ID | Collection | Geographical Origin | Date Collected |
| --- | --- | --- | --- | --- | --- |
| Macrotus waterhousii | 15 | 3123 | AMNH | Mexico: Oxaca | 27 January 1890 |
| Glossophaga soricina valens | 15 | 62404 | AMNH | Ecuador: Duran | 27 April 1922 |
| Molossus molossus crassicaudatus | 15 | 77639 | AMNH | Brazil: Rio Negro - Tatu | 19 September 1928 |
| Molossus molossus molossus | 15 | 175834 | AMNH | Trinidad: Port of Spain | 25 April 1955 |
| Glossophaga longirostris major | 15 | 176606 | AMNH | Tobago: Robinson Crusoe Cave | 04 September 1956 |
| Molossus molossus molossus | 15 | 184746 | AMNH | Tobago: Charlotteville | 31 March 1960 |
| Molossus molossus molossus | 15 | 184747 | AMNH | Tobago: Charlotteville | 31 March 1960 |
| Molossus molossus molossus | 15 | 184750 | AMNH | Tobago: Charlotteville | 31 March 1960 |
| Molossus molossus molossus | 15 | 184754 | AMNH | Tobago: Charlotteville | 31 March 1960 |
| Miniopterus australis tibialis | 15 | 193070 | AMNH | New Guinea: Okapa | 30 September 1959 |
| Carollia perspicillata | 15 | 233502 | AMNH | Peru: Cerros del Sira | mid-July 1969 |
| Carollia perspicillata | 15 | 243995 | AMNH | Venezuela: Zulia | 25 February 1976 |
| Carollia perspicillata | 15 | 244622 | AMNH | Bolivia: Villa Tunari | 10 November 1979 |
| Molossus molossus molossus | 15 | 246261 | AMNH | Trinidad: St. Patrick | 09 April 1969 |
| Carollia perspicillata | 15 | 255722 | AMNH | Colombia: Magdalena | 25 May 1983 |
| Nycteris arge | 15 | 257042 | AMNH | Liberia: Nqilima | 11 August 1983 |
| Carollia perspicillata | 15 | 260114 | AMNH | Bolivia: Santa Cruz | 22 September 1984 |
| Noctilio albiventris affinis | 15 | 262420 | AMNH | Bolivia: Pando | 07 August 1986 |
| Carollia perspicillata | 15 | 262463 | AMNH | Bolivia: Pando | 02 August 1986 |
| Carollia perspicillata | 15 | 262464 | AMNH | Bolivia: Pando | 02 August 1986 |
| Lophostoma silvicolum | 15 | 267422 | AMNH | French Guiana: Paracou | 22 July 1993 |
| Pteronotus parnelli mexicanus | 16 | 172022 | AMNH | Mexico: Sonora, Tesia | 17 April 1956 |
| Trachops cirrhosis | 16 | 210683 | AMNH | Bolivia: Grande River Mouth | 02 August 1965 |
| Artibeus jamciensis fallax | 16 | 210950 | AMNH | Bolivia: Santa Cruz | 22 July 1965 |
| Sturnira bidens | 16 | 216114 | AMNH | Peru: Huariuco | 03 August 1968 |
| Sturnira erythroma | 16 | 233531 | AMNH | Peru: Cordillera | 31 July 1968 |
| Tonatia saurophila | 16 | 266045 | AMNH | French Guiana: Paracou | ND |
| Carollia perspicillata | 16 | 8593 | AMNH | Venezuela: Amazonas | ND |
| Glossophaga soricina valens | 16 | 62422 | AMNH | Ecuador: Guayaquil | 21 July 1922 |
| Molossus molossus crassicaudatus | 16 | 77622 | AMNH | Brazil: Rio Negro - Tatu | 19 September 1928 |
| Noctilio albiventris | 16 | 92438 | AMNH | Brazil: Rosarinho | 10 June 1930 |
| Glossophaga leachii | 16 | 148395 | AMNH | Mexico: Oaxaca | 17 January 1951 |
| Molossus molossus molossus | 16 | 184751 | AMNH | Tobago: Charlotteville | 31 March 1960 |
| Carollia perspicillata azteca | 16 | 186368 | AMNH | Mexico: Oaxaca | 08 March 1961 |
| Brachyphylla cavernarum cavernarum | 16 | 188236 | AMNH | Virgin Islands: St. John, Cruz Bay | 27 January 1962 |
| Miniopterus australis tibialis | 16 | 193032 | AMNH | New Guinea: Okapa | 29 September 1959 |
| Brachyphylla cavernarum cavernarum | 16 | 214016 | AMNH | Lesser Antilles, Martinique, ca 6km | 15 March 1967 |
| Carollia perspicillata | 16 | 243994 | AMNH | Venezuela: Zulia | 25 February 2017 |
| Carollia perspicillata | 16 | 244018 | AMNH | Venezuela: Zulia | 21 February 1976 |
| Carollia perspicillata | 16 | 260130 | AMNH | Bolivia: Santa Cruz | 22 September 1984 |
| Carollia perspicillata | 16 | 260145 | AMNH | Bolivia: Santa Cruz | 22 September 1984 |
| Glossophaga soricina | 16 | 265872 | AMNH | Honduras: Cortes | 06 August 1988 |
| Nycteris hispida | 16 | 268365 | AMNH | Central African Republic | 10 July 1998 |
| Rhogeessa minutella | 16 | 131171a | AMNH | Venezuela: Rio Tocuyo | 23 March 1938 |
| Rhogeessa minutella | 16 | 131171b | AMNH | Venezuela: Rio Tocuyo | 23 March 1938 |
| Pipistrellus papuanus | 16 | 192906 | AMNH | New Guinea: Morobe | 15 October 1959 |
| Pipistrellus papuanus | 16 | 193234 | AMNH | New Guinea: Sepik | 14 January 1960 |
| Pipistrellus angulatus | 16 | 193735 | AMNH | PNG: New Britain | 13 December 1959 |
| Syconycteris australis papuana | 16 | 198429 | AMNH | New Guinea: Sepik | August 1966 |
| Hipposideros doriae | 16 | 234175 | AMNH | Malaya: Pahang | 25 February 1970 |
| Platyrrhinus helleri helleri | 16 | 235788 | AMNH | Colombia: Cauca | 25 February 1973 |
| Platyrrhinus helleri incarum | 16 | 255919 | AMNH | Bolivia: Beni | 22 August 1984 |
| Platyrrhinus helleri incarum | 16 | 260224 | AMNH | Bolivia: Santa Cruz | ND |
| Ametrida centurio | 16 | 267973 | AMNH | French Guiana: Paracou | 26 October 1992 |
| Hipposideros larvatus poutensis | 16 | 275559 | AMNH | Vietnam: Lang Son | 11 April 2004 |
| Hipposideros larvatus poutensis | 16 | 275560 | AMNH | Vietnam: Lang Son | 11 April 2004 |
| Hipposideros larvatus poutensis | 16 | 275562 | AMNH | Vietnam: Lang Son | 11 April 2004 |
| Rhogeessa aeneus | 16 | 277699a | AMNH | Belize: Lamanai | 16 April 2010 |
| Rhogeessa aeneus | 16 | 277699b | AMNH | Belize: Lamanai | 16 April 2010 |
| Molossus pretiosus | 17 | 23776 | AMNH | Venezuela: La Guira | ND |
| Noctilio albiventris | 17 | 92437 | AMNH | Brazil: Rosarinho | 10 June 1930 |
| Carollia perspicillata | 17 | 68881 | AMNH | Ecuador: Guayas | 07 September 2022 |
| Carollia perspicillata azteca | 17 | 186367 | AMNH | Mexico: Oaxaca | 08 March 1961 |
| Carollia perspicillata azteca | 17 | 186371 | AMNH | Mexico: Oaxaca | 08 March 1961 |
| Noctilio leporinus mastivus | 17 | 186969 | AMNH | Cuba: Punta Caguanes | 11 May 1905 |
| Saccopteryx bilineata | 17 | 210491 | AMNH | Bolivia: Beni | 26 September 1965 |
| Carollia perspicillata | 17 | 233451 | AMNH | Peru: Santa Rosa | 15 August 1968 |
| Molossus molossus molossus | 17 | 246258 | AMNH | Trinidad: St. Patrick | 11 April 1969 |
| Molossus molossus molossus | 17 | 246259 | AMNH | Trinidad: St. Patrick | 11 April 1969 |
| Molossus molossus molossus | 17 | 246263 | AMNH | Trinidad: St. Patrick | 09 April 1969 |
| Glossophaga soricina | 17 | 247963 | AMNH | Bolivia: El Beni | 30 August 1980 |
| Carollia perspicillata | 17 | 255730 | AMNH | Colombia: Magdalena | 08 June 1983 |
| Carollia perspicillata | 17 | 260134 | AMNH | Bolivia: Santa Cruz | 22 September 1984 |
| Molossus molossus crassicaudatus | 17 | 260279 | AMNH | Bolivia: Santa Cruz | 04 October 1984 |
| Rhogeessa minutella | 17 | 131188a | AMNH | Venezuela: Rio Tocuyo | 24 March 1938 |
| Rhogeessa minutella | 17 | 131188b | AMNH | Venezuela: Rio Tocuyo | 24 March 1938 |
| Pipistrellus papuanus | 17 | 192907 | AMNH | New Guinea: Morobe | 15 October 1959 |
| Rhinolophus coelophyllus | 17 | 216854 | AMNH | Malaya: Kedah | 03 March 1968 |
| Syconycteris australis papuana | 17 | 221843 | AMNH | New Guinea: Irian Jaya | 01 December 1961 |
| Platyrrhinus dorsalis | 17 | 233615 | AMNH | Peru: Cordillera | 31 July 1968 |
| Platyrrhinus dorsalis | 17 | 233640 | AMNH | Peru: Cerro del Sira | 19 July 1969 |
| Hipposideros larvatus neglectus | 17 | 234166 | AMNH | Malaya: Kedah | 23 March 1970 |
| Epomophorus gambianus | 17 | 237405 | AMNH | Ghana: Mole Natl Park | 10 July 1974 |
| Platyrrhinus helleri incarum | 17 | 255918 | AMNH | Bolivia: Beni | ND |
| Hipposideros larvatus poutensis | 17 | 275561 | AMNH | Vietnam: Lang Son | 11 April 2004 |
| Hipposideros larvatus poutensis | 17 | 275565 | AMNH | Vietnam: Lang Son | 11 April 2004 |
| Rhinolophus pearsonii | 17 | 275621 | AMNH | Vietnam: Lang Soni | 18 April 2004 |
| Phyllostomus discolor | 17 | 209326 | AMNH | Bolivia: Guayaramarin | 30 June 1964 |
| Monophyllus redmani | 17 | 217779 | AMNH | Jamaica: St. Catherine Parish, St. Clair Cave | 29 December 1965 |
| Artibeus glaucus | 17 | 233778 | AMNH | ND | ND |
| Monophyllus redmani | 17 | 271777 | AMNH | Jamaica: St. Catherine Parish, St. Clair Cave | 29 December 1965 |
| Monophyllus redmani | 17 | 271783 | AMNH | Jamaica: St. Catherine Parish, St. Clair Cave | 29 December 1965 |
